# The mechanism of fibronectin 1 promoting papillary thyroid cancer progression by regulating anoikis resistance

**DOI:** 10.1038/s41598-026-43495-8

**Published:** 2026-04-19

**Authors:** Shengcan Zhang, Xue Gu, Xinyu Zhai, Hanzhao Huang, Renjie Zhang, Hui Ye

**Affiliations:** 1 Clinical Medical College, Guizhou Medical University, Guiyang, 551113 China; 2 Thyroid Surgery Department, The Affiliated Hospital of Guizhou Medical University, Guiyang, 550004 China

**Keywords:** Fibronectin 1, Papillary thyroid cancer, Anoikis resistance, Tumor progression, Biomarkers, Cancer, Computational biology and bioinformatics, Oncology

## Abstract

Background: Papillary thyroid cancer is a common head and neck malignancy with a globally rising incidence. While most patients attain favorable prognosis following standard treatment, a subset develops recurrence and metastasis—two key factors that compromise clinical outcomes. Anoikis resistance is a prerequisite for tumor metastasis; however, the role and mechanism of Fibronectin 1 (FN1)—a molecule overexpressed in multiple cancers and linked to tumor progression—in papillary thyroid cancer anoikis resistance remain undefined. Objective: This study therefore investigated the function and regulatory mechanism of FN1 in papillary thyroid cancer anoikis resistance using a combination of bioinformatics analyses and experimental validation. Methods: First, using The Cancer Genome Atlas (TCGA) dataset and anoikis-related genes from GeneCards, we identified FN1 as an anoikis-related hub gene in thyroid cancer through bioinformatics analyses, including consensus clustering, weighted gene co-expression network analysis (WGCNA), and protein-protein interaction (PPI) network analysis. Its clinical relevance was further analyzed from the perspectives of pathological typing, survival prognosis, immune cell infiltration (via xCell) and single-cell expression patterns. Next, we performed validation using papillary thyroid cancer tissues, adjacent normal tissues, and cell lines (8305c, BCPAP, Nthy-ori 3 − 1): FN1 expression and cancer cell anoikis were detected by immunohistochemistry (IHC) and TUNEL staining; FN1 mRNA and protein levels were quantified via qRT-PCR and Western blot. Finally, we modulated FN1 expression through RNA interference (for knockdown). Cell proliferation, migration, and invasion were assessed using CCK-8 and Transwell assays, while Western blot was applied to measure the expression of apoptosis-related proteins. Results: FN1 is highly expressed in papillary thyroid cancer tissues and cell lines and is associated with poor tumor prognosis. Silencing FN1 can inhibit the proliferation, migration and invasion of papillary thyroid cancer cells and increase the anoikis rate. Overexpression of FN1 showed the opposite effect. Mechanistically, FN1 can up-regulate anti-apoptotic proteins BCL2L1 and Caspase-3, and down-regulate pro-apoptotic protein BAD, thereby enhancing anoikis resistance. Conclusions: FN1 is highly expressed in papillary thyroid cancer and correlates with poor prognosis. Mechanistically, FN1 enhances papillary thyroid cancer cell tumorigenic potential by regulating proliferation, migration, invasion and apoptotic progression via the BCL2L1/BAD axis, thus driving tumor development. FN1 may act as a promising therapeutic target for advanced papillary thyroid cancer.

**Supplementary Information:**

The online version contains supplementary material available at 10.1038/s41598-026-43495-8.

## Introduction

Papillary papillary thyroid cancer (PTC) is a rapidly growing endocrine malignancy worldwide; the increase in its incidence is mainly due to the improvement of high-resolution ultrasound and molecular cytology detection techniques, which has also brought a certain burden to global public health^1, 2^. While most indolent cases achieve favorable prognoses following curative surgery, a subset of patients develop recurrence and metastasis, which represent the leading cause of mortality in this disease^3^, and such advanced or metastatic lesions are often refractory to conventional therapies, including radioiodine treatment and systemic chemotherapy^1^. Current risk stratification and treatment frameworks fail to address the biological heterogeneity that drives disease progression^3^, and targeted therapies as well as active surveillance strategies yield only modest benefits for patients with aggressive or treatment-resistant phenotypes^2^, creating an urgent need to elucidate the molecular determinants underlying metastatic potential and therapeutic resistance in thyroid malignancies to elucidate the molecular determinants that drive metastatic potential and therapeutic resistance within thyroid malignancies.

One key biological process implicated in tumor dissemination is anoikis resistance, a form of apoptosis evasion that enables malignant cells to survive detachment from the extracellular matrix during metastatic spread. Anoikis resistance is increasingly recognized as a hallmark of metastatic competence in various epithelial cancers, facilitating both local invasion and colonization of distant sites. Recent research has highlighted the role of the tumor microenvironment and extracellular matrix (ECM) components in modulating this phenotype, with several ECM glycoproteins and their associated signaling pathways identified as central mediators^4^.

**Fig. 1:**
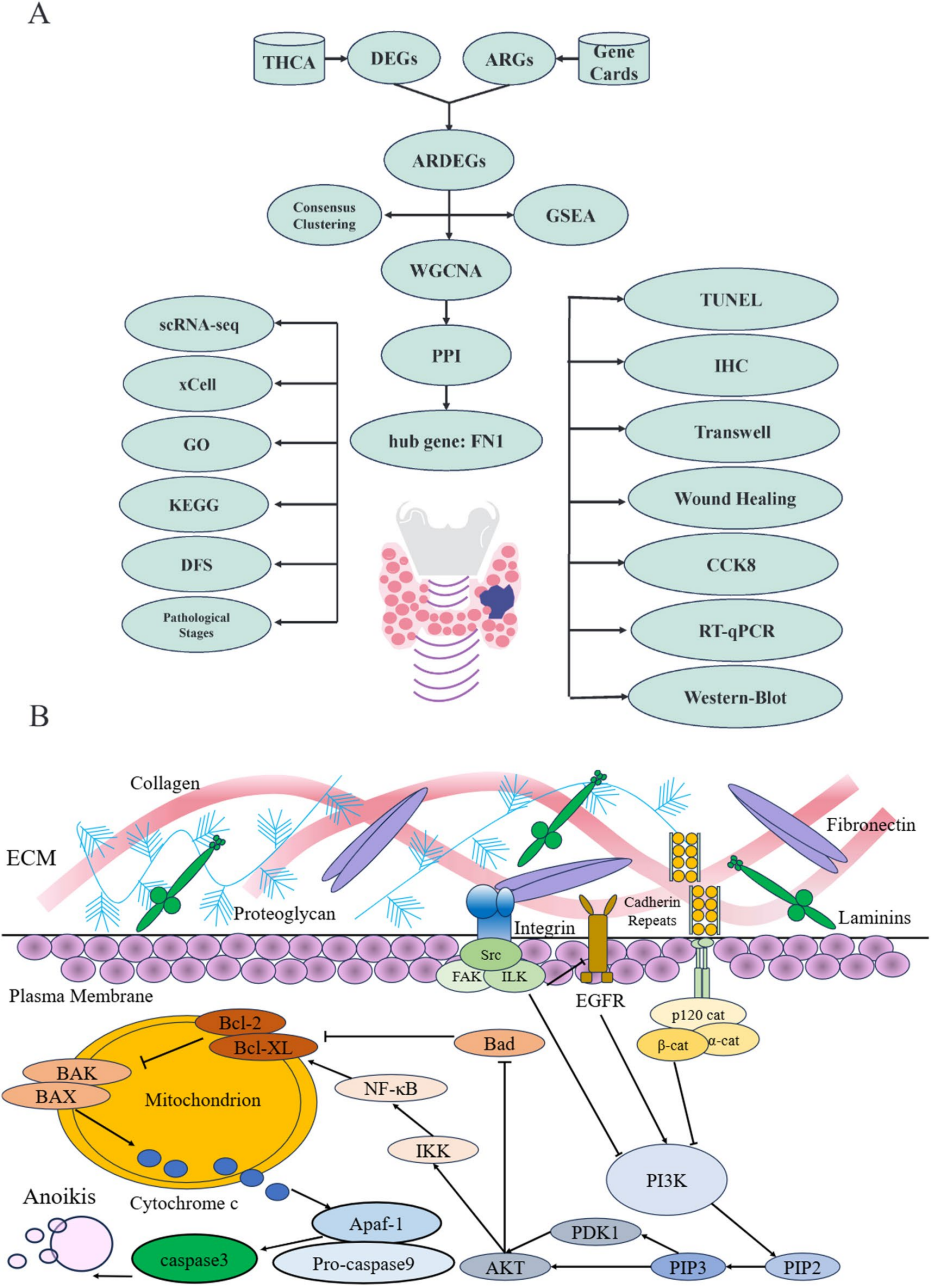
(**A**) Flowchart of this study. (**B**) Activation of anoikis pathways. Lack of ECM contact or the engagement with inappropriate ECM fails to activate pro-survival signals leading to the decrease of anti-apoptotic pathways, thus activating anoikis from death receptors and mitochondria.

Fibronectin 1 (FN1), as a major ECM protein, has been shown to be overexpressed in multiple solid tumors. As a pivotal extracellular matrix glycoprotein, FN1 exerts its pro-tumorigenic functions by binding to integrin family receptors located on the cell surface, which in turn triggers the phosphorylation and activation of the downstream non-receptor tyrosine kinase FAK. In the interaction between cells and ECM, the Integrin/FAK signaling axis binds closely to ECM through integrin-linked kinase; this binding can activate focal adhesion kinase FAK and induce its conformational changes, leading to the aggregation of the intracellular domain of FAK^5^. This aggregation process not only recruits and activates FAK, but also promotes the phosphorylation of tyrosine residues of FAK, thereby fully activating FAK. Activated FAK can form multiple phosphorylated tyrosine sites, which serve as binding sites for signaling molecules to further recruit and activate downstream signaling pathways, including the PI3K/AKT, ERK, and JNK pathways^6^. Notably, activated FAK can also phosphorylate Bcl-2-associated death promoter (BAD), prompting its dissociation from the anti-apoptotic protein Bcl-XL to exert a significant anti-apoptotic effect; in addition, FAK can activate Src-family kinases (SFKs), which exert extensive regulatory effects in cells—they not only regulate multiple signaling pathways, but also inhibit the function of pro-apoptotic proteins by regulating the activity of Bcl-2 family proteins, and stabilize cell morphology by regulating the reorganization of cytoskeletal proteins, enabling cells to survive in the adverse environment of detachment from ECM^7,8^ (Fig. 1B). Activated FAK further propagates intracellular signaling by phosphorylating Akt, governing a wide spectrum of cellular processes essential for tumor progression, including cell proliferation, survival, anti-apoptotic capacity, migration, and invasion.

However, the specific mechanistic contribution of FN1 to anoikis resistance in papillary thyroid cancer remains largely unexplored, representing a significant gap in current knowledge.

To address this gap, the present study integrates large-scale bioinformatics analyses with experimental validation to systematically investigate the function and regulatory mechanisms of FN1 in papillary thyroid cancer, particularly in the context of anoikis resistance. Leveraging publicly available genomic datasets, such as The Cancer Genome Atlas (TCGA), in conjunction with curated gene sets related to anoikis from resources like GeneCards, enables a comprehensive exploration of FN1 expression patterns, their correlation with clinicopathological features, and association with patient outcomes^9^. Furthermore, through the application of advanced computational approaches—including consensus clustering, weighted gene co-expression network analysis (WGCNA), and protein-protein interaction (PPI) network modeling—this study aims to delineate the centrality of FN1 within the anoikis regulatory network of papillary thyroid cancer^10^. The integration of in silico findings with in vitro functional assays permits a robust assessment of FN1’s biological relevance and its potential as a novel therapeutic target.

The methodological framework adopted herein is distinguished by the combination of high-dimensional data mining, multi-omics integration, and rigorous laboratory experimentation (Fig. 1A). This approach not only facilitates the identification of key molecular drivers, such as FN1, but also enables the interrogation of their downstream effectors and associated signaling pathways relevant to cell survival, apoptosis, and immune microenvironment modulation^4,11^. Such a comprehensive design ensures that candidate targets are evaluated in both computational and biological systems, enhancing the translational value of the findings. Moreover, the study leverages state-of-the-art bioinformatics pipelines and leverages immune infiltration analyses to further contextualize FN1’s role within the tumor-immune landscape, which has become increasingly pertinent in the era of immunotherapy^11^.

In summary, this research seeks to elucidate the role of FN1 in mediating anoikis resistance in papillary thyroid cancer and to unravel the underlying regulatory mechanisms through an integrative strategy encompassing bioinformatics and experimental validation. By systematically characterizing the clinical and biological significance of FN1, the study aims to provide foundational evidence for its utility as a prognostic biomarker and a promising therapeutic target for advanced or refractory papillary thyroid cancer. The anticipated insights from this investigation are poised to inform the development of more precise and effective intervention strategies, ultimately contributing to improved patient stratification and clinical outcomes in thyroid oncology (Fig 1).

## Methods

### Public database analysis

To systematically characterize the expression profile, clinical relevance, and regulatory network of FN1 in thyroid cancer, multi-dimensional bioinformatics analyses were conducted using publicly available omics datasets, with detailed protocols as follows:

#### Data acquisition and preprocessing

TCGA-THCA Dataset: Level 3 RNA-sequencing (RNA-seq) data (FPKM format) and matched clinical metadata (including pathological stage, TNM classification, and survival outcomes) of thyroid carcinoma (THCA) were retrieved from The Cancer Genome Atlas (TCGA) database (https://portal.gdc.cancer.gov/), encompassing 510 primary thyroid cancer samples and 58 adjacent non-tumor thyroid samples. Raw FPKM values were normalized to Transcripts Per Million (TPM) using the DESeq2 package in R (v4.3.2) to eliminate biases arising from differences in sequencing depth. Samples with incomplete clinical information (e.g., missing TNM stage or survival time) were excluded, yielding a final cohort of 498 qualified cancer samples for subsequent analyses.

Anoikis-Related Genes (ARGs): A total of 908 ARGs were obtained from the GeneCards database (https://www.genecards.org/) using “anoikis” as the search term. To ensure functional relevance to anoikis regulation, only genes with a “Relevance Score” ≥ 50 were retained for further analysis.

Single-Cell RNA-Seq (scRNA-seq) Dataset: A public scRNA-seq dataset (GSE 241184) was downloaded from the Gene Expression Omnibus (GEO) database (https://www.ncbi.nlm.nih.gov/geo/), which contains 16,897 cells derived from 1 Patients with.

Thyroid carcinoma and lymph node metastasis. Quality control was performed using the Seurat package (v4.9.9.9000): cells with mitochondrial gene content > 5% (indicating potential cell damage) or unique gene counts < 200 (indicating low-quality cells) were filtered out. The SCTransform function was applied for normalization and batch effect correction to minimize technical variability between samples.

GEPIA Database Validation: The GEPIA database (http://gepia.cancer-pku.cn/), a web-based tool for cancer and normal gene expression profiling, was used to validate the differential expression of FN1 between thyroid cancer and normal tissues, as well as its association with disease-free survival (DFS). Analyses were conducted using the “Expression DIY” and “Survival Analysis” modules, with statistical significance determined by the log-rank test (*P* < 0.05).

#### Identification of differentially expressed anoikis-related genes

Differential expression analysis between TCGA-THCA cancer samples and adjacent non-tumor samples was performed using the limma package in R. Genes were defined as differentially expressed genes (DEGs) if they met the criteria: |log₂FoldChange| > 1 and adjusted P-value (false discovery rate, FDR) < 0.05. A Venn diagram was constructed using the VennDiagram package to intersect DEGs with ARGs, resulting in 196 differentially expressed anoikis-related genes (DE-ARGs). A volcano plot was generated using the ggplot2 package to visualize the distribution of upregulated (red dots) and downregulated (blue dots) DE-ARGs.

#### Anoikis-related subgroup clustering and survival analysis

Unsupervised consensus clustering was performed on the 498 TCGA-THCA samples using the ConsensusClusterPlus package, based on the expression matrix of 196 DE-ARGs. The optimal number of clusters (k) was determined by evaluating the consensus cumulative distribution function (CDF) curve and delta area plot: the clustering stability reached its maximum when k = 2, thus samples were divided into two anoikis-related subgroups (Subgroup 1 and Subgroup 2). A heatmap was generated using the pheatmap package to visualize the expression patterns of DE-ARGs across the two subgroups, with the risk table and log-rank test P-value supplemented below the survival curve. Kaplan-Meier survival curves were constructed using the survminer and survival packages to compare DFS between the two subgroups, with statistical significance assessed by the log-rank test (*P* < 0.05).

#### Weighted gene co-expression network analysis (WGCNA) and hub gene identification

WGCNA Construction: WGCNA was performed using the WGCNA package in R to construct a gene co-expression network. The optimal soft threshold power (power = 7) was selected based on the scale-free topology criterion (R² = 0.85), ensuring the network conformed to scale-free characteristics. Genes were clustered using hierarchical clustering, and the dynamic tree cutting method (minModuleSize = 30, mergeCutHeight = 0.25) was used to partition genes into four co-expression modules (turquoise, brown, blue, and gray; the gray module represents unassigned genes with no clear co-expression pattern).

Module-Trait Correlation: The correlation between each module’s eigengene (a representative gene for the module) and clinical traits (stromal score, immune score, ESTIMATE score, and tumor purity, calculated using the ESTIMATE package) was analyzed. The turquoise module was identified as the most strongly associated with tumor purity (*r* = 0.5, *P* = 2.3 × 10⁻⁶), and 80 core genes were extracted from this module for subsequent hub gene screening.

Protein-Protein Interaction (PPI) Network and Hub Gene Screening: The 80 core genes from the turquoise module were uploaded to the STRING database (v11.5, https://string-db.org/) to construct a PPI network, with the minimum interaction confidence score set to 0.7 (high confidence). The network was visualized using Cytoscape software (v3.10.2), and the “CytoHubba” plugin was used to identify hub genes based on the Degree algorithm (ranking genes by the number of interacting partners). FN1 was identified as the top hub gene (Degree = 37), confirming its central role in the anoikis regulatory network of thyroid cancer.

#### Functional enrichment and immune infiltration analysis

Functional Enrichment Analysis: FN1-related genes were defined as genes with a Pearson correlation coefficient |r| > 0.4 with FN1 in the TCGA-THCA dataset. Gene Ontology (GO) enrichment analysis (including biological processes [BP], cellular components [CC], and molecular functions [MF]) and Kyoto Encyclopedia of Genes and Genomes (KEGG) pathway enrichment analysis were performed on these FN1-related genes using the clusterProfiler package in R. The complete tables of GO and KEGG enrichment analyses, including all statistical data such as adjusted P-value and FDR, have been uploaded to the Zenodo platform. Enrichment results were visualized using bubble plots, with statistical significance set at adjusted *P* < 0.05.

Immune Infiltration Analysis: The xCell algorithm (v1.0.2), implemented via the xCell package in R, was used to calculate the relative infiltration abundance of 64 immune cell types in the TCGA-THCA samples. Samples were stratified into FN1-high and FN1-low groups based on the median TPM value of FN1. The Wilcoxon rank-sum test was used to compare differences in immune cell infiltration between the two groups, and a heatmap was generated to visualize the distribution of anti-tumor immune cells (e.g., CD8⁺ T cells, natural killer cells) across groups.

#### Single-cell level expression analysis of FN1

Using the preprocessed GSE183227 scRNA-seq dataset, t-distributed stochastic neighbor embedding (tSNE) dimensionality reduction was performed using the Seurat package to reduce the dimensionality of the single-cell data. Cell types were annotated based on canonical marker genes: EPCAM for epithelial cells, CD3D for T cells, CD68 for macrophages, CD31 for endothelial cells, and SOX9 for tissue stem cells. The FeaturePlot function in Seurat was used to map the expression level of FN1 onto the tSNE plot, with red indicating high FN1 expression and blue indicating low expression, to identify the primary cell populations expressing FN1 in thyroid cancer and normal tissues.

### Clinical study

#### Clinical tissue specimens

This study recruited 33 PTC patients (batch inclusion: 18 cases in the first batch and 15 cases in the subsequent supplementary batch) who underwent resection at the Affiliated Hospital of Guizhou Medical University from November 2024 to August 2025. The key criteria of exclusion for participants were a cognitive impairment, received preoperative chemoradiotherapy and concomitant with other malignancies. The inclusion criteria: age: 30–70 years old, initial treatment (first-time treatment for the disease) and pathologically confirmed papillary thyroid carcinoma (PTC). The whole process was supervised by the Ethics Review Committee of the Affiliated Hospital of Guizhou Medical University, and strictly abides by the ‘Helsinki Declaration’. The ethics review approval number was Ethics Approval No: GMU2024-452. All participants submitted informed consent prior to tissue sampling. The details are presented in Online Supplementary material 1. Among the 33 PTC patients, 16 cases (from the first batch of 18 cases with complete paired tissues and qualified section quality) were selected as the TUNEL detection subcohort; the remaining 2 cases of the first batch had section shedding during sample preparation, and the 15 newly supplemented cases were limited by experimental timing and reagents, so they were not included in the TUNEL subcohort. Finally, a total of 33 participants were enrolled, of which 32 cases of papillary thyroid cancer tissue, 32 cases of thyroid peritumoral tissue and 1limphy node tissue.

### Cell experiments

#### Cell culture

The 8305c cells used in this study were obtained from ProCell Life Science and Technology (Wuhan, China). The 8305 C cells were cultured in MEM medium (supplemented with 2 mM L-glutamine), with the addition of 10% fetal bovine serum (FBS) and 1% penicillin-streptomycin (P/S) dual antibiotic. The culture was maintained under the following conditions: temperature of 37 °C (± 0.5 °C) and 5% CO₂ concentration. The BCPAP cells used in this study were obtained from ProCell Life Science and Technology (Wuhan, China). The BCPAP cells were cultured in DMEM medium (supplemented with 2 mM L-glutamine), with the addition of 10% fetal bovine serum (FBS) and 1% penicillin-streptomycin (P/S) dual antibiotic. The culture was maintained under the following conditions: temperature of 37 °C (± 0.5 °C) and 5% CO₂ concentration.

#### Cell transfection

The FN1-siRNA (si-FN1) and negative control (si-NC) siRNAs were procured from Sangon Biotech, China. FN1 siRNA (5’-CGG UUG UUA UGA CAA UGG A-3’ (dT)(dT))and negative control (NC) siRNA (a non-targeting scrambled siRNA that does not target any known human gene and shares no homology with FN1) were transfected into cells and then incubated for 6 h. CLANPTMRNA in vitro (D-Nano Therapeutics, China) was used to facilitate the siRNA transfection process, strictly adhering to the manufacturer’s instructions. To verify the effectiveness of the siRNA, RealTime Polymerase Chain Reaction (qRT-PCR) was performed 24 h following transfection to evaluate FN1 expression levels, and only the cells with FN1 knockdown efficiency > 70% were used for subsequent functional experiments.

#### CCK-8 assay

Cell viability was evaluated using the CCK-8 assay. Cells were seeded in 96-well plates at a density of 3 × 10³ cells per well, with 5 replicate wells set for each group. After incubation for 0, 24, and 48 h in a 37 °C, 5% CO₂ incubator, respectively, 10 µL of CCK-8 solution (Biosharp, China) was added to each well. Following incubation for an additional 0.5–1 h in 36 °C, the optical density (OD) at 450 nm was measured using a microplate reader, and the experiment was independently repeated 3 times.

#### Immunohistochemical analysis

The slides were treated with 0.3% hydrogen peroxide (H₂O₂) prepared in methanol for 30 min to block endogenous peroxidase activity, followed by permeabilization with 0.5% Triton X-100 for 30 min at room temperature; antigen retrieval was then performed by incubating the sections in citrate buffer (pH 6.0) at the appropriate working strength using a microwave or pressure cooker according to standard protocols, and after cooling to room temperature and washing three times with PBS, the slides were blocked with 5% normal goat serum for 30 min to prevent nonspecific binding. Primary antibodies against FN1 (Proteintech, 15613-1-AP, China) were diluted at 1:5000 in antibody dilution buffer and incubated with the sections for 2 h at room temperature; after three washes with PBS, the slides were incubated with HRP-conjugated anti-rabbit IgG (H + L) secondary antibody (1:500 dilution in PBS containing 1% BSA) for 30 min at room temperature, followed by three additional PBS washes, visualization of immunoreactivity using 3,3’-diaminobenzidine (DAB) for 5 min with monitoring under a microscope to ensure optimal signal-to-noise ratio, and termination of the reaction by rinsing with distilled water. The nuclei were counterstained with hematoxylin for approximately 30 s, followed by dehydration, clearing, and mounting with neutral balsam; for immunofluorescence detection, DAPI was used for nuclear counterstaining for 5 min, and slides were mounted with anti-fade mounting medium.

Immunostaining results were independently evaluated by two pathologists in a double-blinded manner, with discrepancies resolved by consensus or by a third pathologist, and scored based on the percentage of positive cells as follows: negative (< 10%), weakly positive (10–50%), and strongly positive (> 50%), or using the H-score calculated as H-score = Σ (PI × I), where PI is the percentage of positive cells (0–100%) and I is the staining intensity (0 = negative, 1 = weak, 2 = moderate, 3 = strong), with the total score ranging from 0 to 300, and all experiments were repeated at least three times independently.

#### Transwell migration and invasion assay

Cell migration and invasion were assessed using Transwell assays. For both assays, cells were seeded into the upper chamber of 24-well Transwell inserts (8-µm pore size, NEST, China) containing serum-free medium; the lower chamber was filled with medium supplemented with 20% FBS as a chemoattractant. For invasion assays, the upper surface of the membrane was pre-coated with 50 µL of Matrigel (1 mg/mL) at 4 °C overnight and rehydrated with serum-free medium before cell seeding. After 1 days, the cells having not migrated through the chamber membrane were removed, and those having passed through the membrane were subjected to fixation and staining with crystal violet. They were subsequently detected and photographed using a microscope.

#### Wound-healing assay

Wound-Healing Assay was conducted to assess thyroid cancer cells’ (8305c, BCPAP) and normal thyroid cell (Nthy-ori 3 − 1) migratory capacity: Cells were seeded in 6-well plates, cultured in 10% FBS-supplemented DMEM/MEM to 90%-95% confluency; FN1-regulated cells were transfected with si-FN1/si-NC 48 h prior (CLANPTMRNA in vitro) and FN1 knockdown verified by qRT-PCR/Western blot. A uniform straight wound was made in each well with a sterile 200 µL pipette tip, wells rinsed twice with PBS to remove debris, medium replaced with serum-free DMEM/MEM, and plates incubated at 37°C with 5% CO₂. Wound area images were captured at 0 h and 24 h (100×, inverted phase-contrast microscope), three random fields per well photographed; wound width was measured via ImageJ to calculate scratch healing rate, with the experiment independently repeated three times (three replicates per group).

### Validation of key targets

#### qRT-PCR

MolPure^®^ Cell/Tissue Total RNA Kit (Yeasen, China) was used to extract total RNA. Reverse Transcriptase Kit (Yeasen, China) was used for performing reverse transcription reactions at 42°C. qRT-PCR analyses were conducted using SYBR Green supermix (Yeasen, China) according to a two-step procedure. GAPDH was used for normalizing gene expressions. ΔΔCt or 2-ΔΔCt method was applied for data analysis. Synthesis of primers was conducted in Sangon Biotech (Shanghai, China). (Supplementary material 3)

#### Western blot

Extraction of total proteins was performed with RIPA lysis buffer containing protease inhibitors. Proteins were detected using a BCA protein assay kit (Servicebio, China) prior to being separated by SDS-PAGE and transferred onto PVDF membranes (Servicebio, China). The membranes were then subjected to incubation with the following primary antibodies at the indicated dilutions: anti-FN1 (Servicebio: GB152093, China) at a dilution of 1:1000, anti-BAD (Servicebio: GB113746, China) at a dilution of 1:1000, anti-Caspase-3 (Servicebio: GB11767C, China) at a dilution of 1:1000, anti-BCL2L1/Bcl-xl (Proteintech: 10783-1-AP, China) at a dilution of 1:1000, and anti-GAPDH (Servicebio, China) at a dilution of 1:10000. After washing, the membranes were incubated with HRP-conjugated secondary antibodies at room temperature for 1 h. The signal was examined with an AIWBwellTM (Servicebio, China) as specified by the vendor.

### Statistical analysis

All data were analyzed by SPSS 26.0. Continuous data were presented as mean ± SD. Differences between two groups were compared by t-test; multiple groups by one-way ANOVA. Survival analysis was by Kaplan-Meier with log-rank test. *P* < 0.05 was considered statistically significant.

## Results

### Public database analysis

#### Identification of differentially expressed anoikis-related genes

**Fig. 2:**
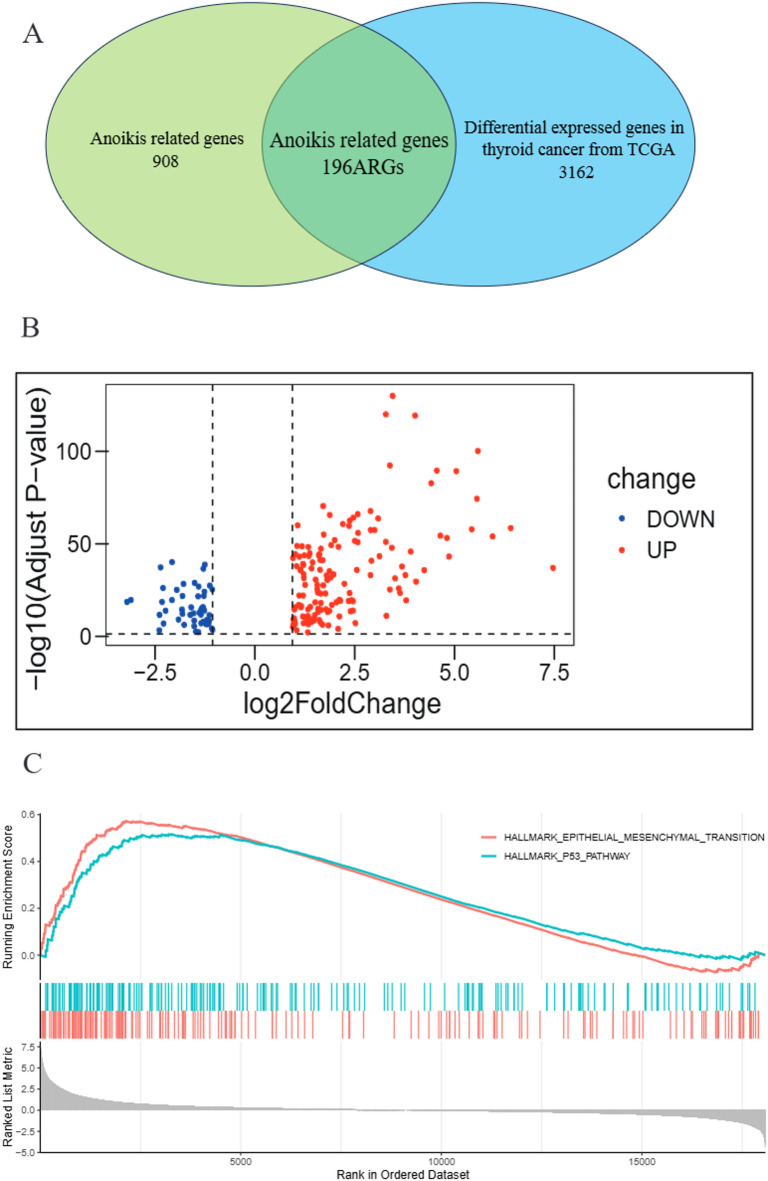
Identification of differentially expressed anoikis-related genes. (**A**) Venn diagram of DE-ARGs in TCGA and anoikis-related gene. (**B**) Volcano plot of differentially expressed genes in the TCGA dataset. Blue dots indicate statistically significant downregulated genes and red dots indicate statistically significant upregulated genes. The x-axis represents the logarithm of fold change of differentially expressed genes and the y-axis represents the adjusted p-value based on the FDR correction met. (**C**) GSEA summary plot showing enrichment of p53 pathways and EMT in ARGs. X-axis: ranked genes in dataset; Y-axis: running enrichment score (RES).

In order to identify differentially expressed anoikis-related genes (DE-ARGs), we obtained 3162 differentially expressed genes (DEGs) from thyroid cancer (THCA) samples obtained from the TCGA database (Fig. 2A). Then, we found 908 anoikis-related genes from the Gene Cards database, and finally screened 196 differentially expressed differentially expressed anoikis-related genes for further analysis (Fig. 2B). Through GSEA analysis, we found that thyroid cancer-related anoikis genes were mainly enriched in epithelial-mesenchymal transition and P53 pathway (Fig. 2C).

#### Identification of anoikis-related subgroups

**Fig. 3:**
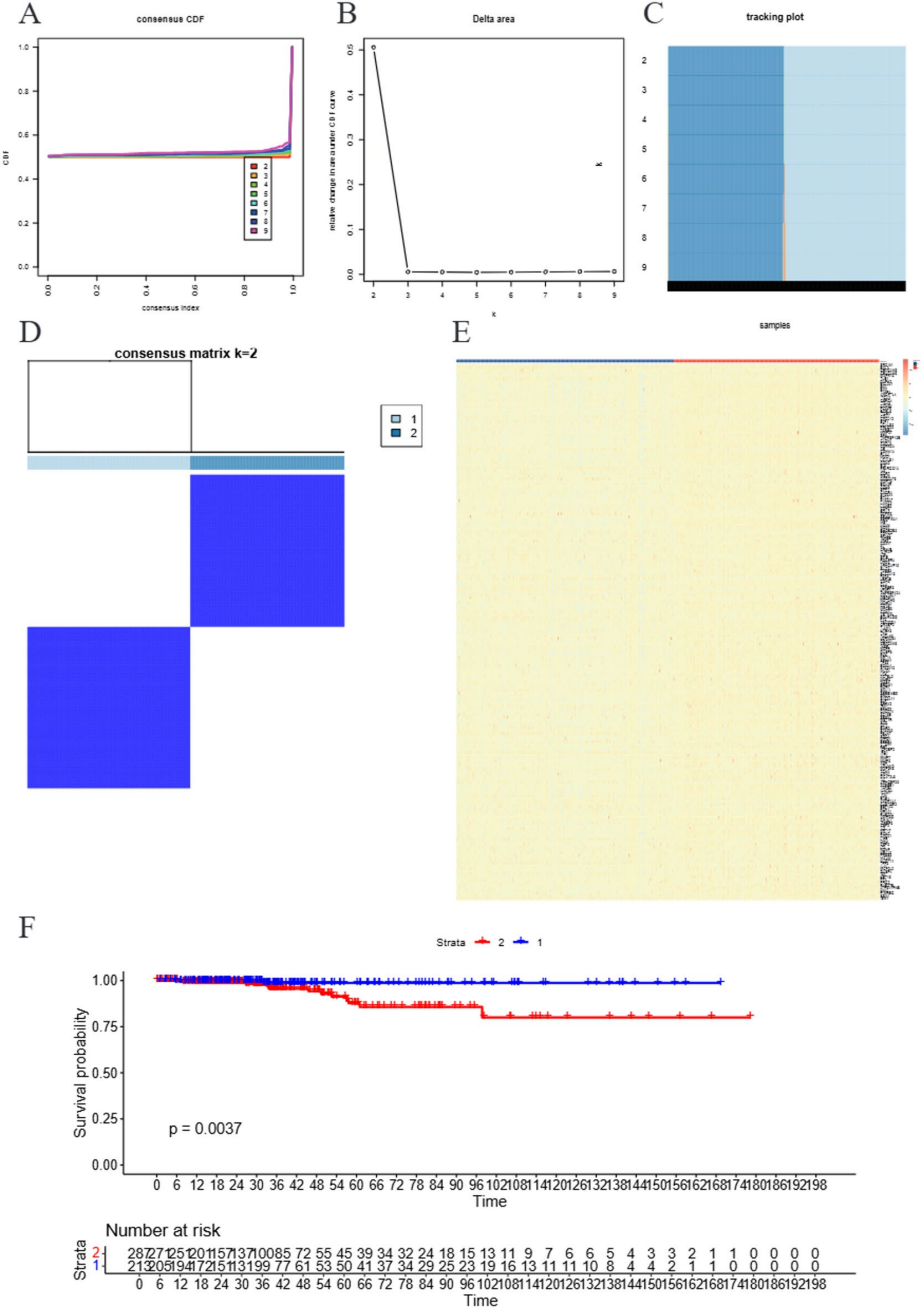
Identification of Anoikis-Related Subgroups. (**A**) Consensus cumulative distribution function (consensus CDF), showing the cumulative distribution characteristics corresponding to different consistency indexes. (**B**) Delta area diagram, reflecting the change trend of Delta area when the number of clusters changes. (**C**) Tracking plot, showing the tracking result of the sample in the clustering process. (**D**) Consensus matrix (consensus matrix k = 2 ) when the number of clusters k = 2, the blue block represents the consistency grouping of clustering between samples. (**E**) Heat map, showing the feature distribution of samples in time and other dimensions. (**F**) Kaplan-Meier survival curve, compare the survival probability of group 1 and strata 2 ( log-rank test *p* = 0.0037 ), below is the number of people at risk at different time points.

To identify anoikis-related subgroups, a common clustering algorithm was applied for unsupervised clustering to explore potential clusters in the TCGA-THCA dataset, with the analysis based on the expression profiles of DE-ARGs with |log₂FoldChange| > 1 and adjusted P-value (false discovery rate, FDR) < 0.05. The results show that when k = 2 is the best parameter to divide the data set into 1 and 2 subgroups (Fig. 3A–D). The expression heat map of DE-ARGs showed differences between the two subgroups (Fig. 3E). Moreover, the survival probability of subgroup 2 was lower than that of subgroup 1 (Fig. 3F). These results suggest that TCGA-THCA can be classified according to anoikis-related genes.

#### Identification of highly correlated gene module in TCGA-THCA

**Fig. 4:**
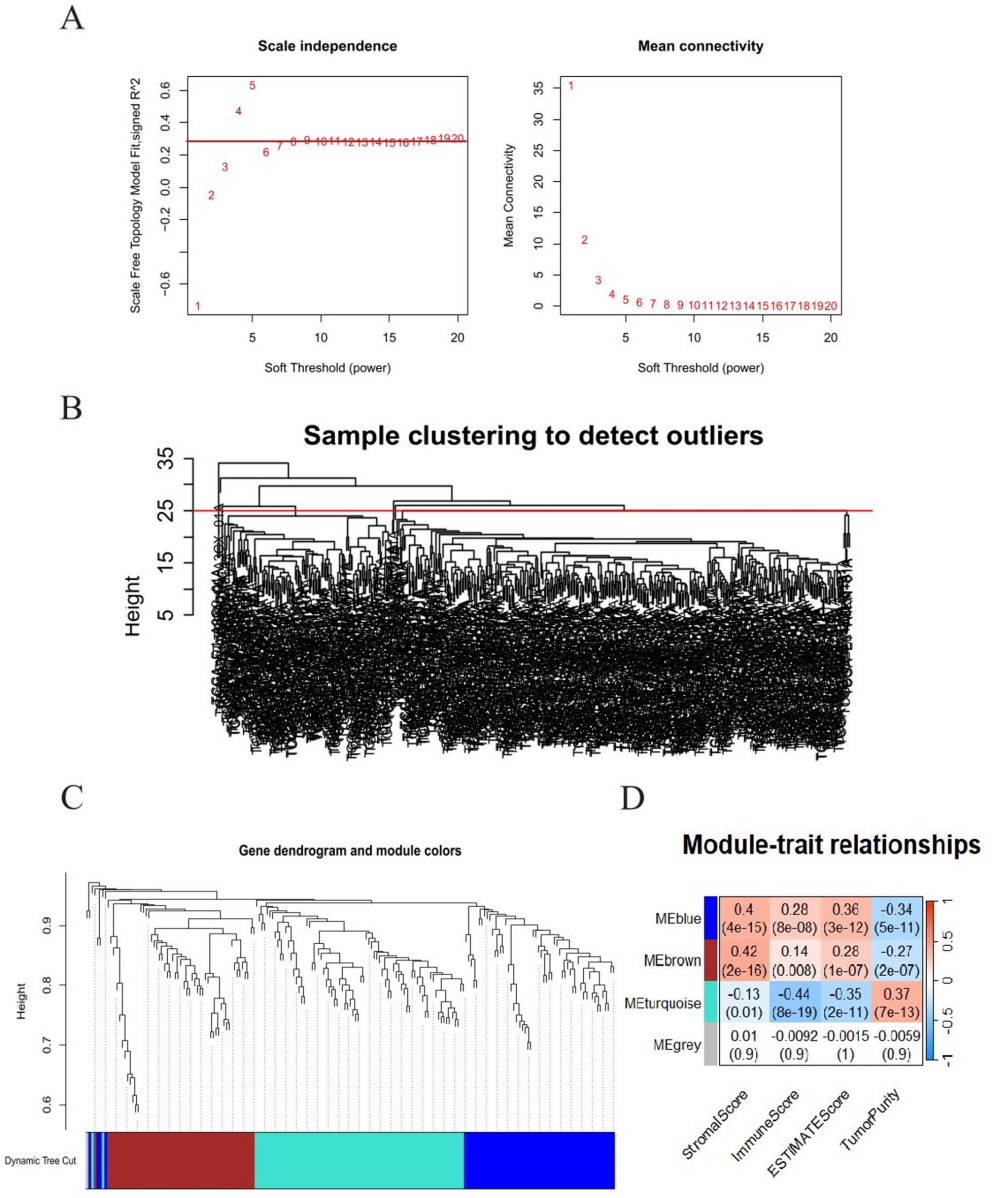
Identification of Highly Correlated Gene Module in TCGA-THCA. (**A**) soft threshold determination analysis. The left figure is the scale independence test (showing the fitting index *R*^*2*^ of thescale-free topological model under different soft thresholds), and the right figure is the average connectivity analysis (showing the average connectivity under different soft thresholds). (**B**) Sample clustering tree diagram, which is used to detect outlier samples, and the red horizontal line is the reference line for outlier determination. (**C**) Gene phylogenetic tree and module color. The results of gene clustering into different modules are shown by dynamic shear tree. (**D**) module-phenotypic trait association heat map, showing the correlation coefficients between each gene module and StromalScore, ImmuneScore, ESTIMATEScore, TumorPurity (*P* values in parentheses). (**E**) The scatter diagram of the relationship between the module members of the genes in the turquoise module and the significance of the genes related to the tumor purity (*R*^*2*^ = 0.43, *P* = 4.8 × 10 − 6). (**F**) Protein interaction network, nodes represent genes, showing the connection relationship of hub genes.

In order to explore the highly correlated genes in DE-ARGs, we performed WCGNA to identify highly correlated gene modules (Fig. 4B). Then, we set the soft threshold power to 7 (Fig. 4A), and identified four gene modules according to the gene tree: turquoise module, brown module, gray module and blue module (Fig. 4C). In these gene modules, Tumor purity was strongly correlated with the turquoise module (*r* = 0.5, *P* = 2.3 × 10⁻⁶) (Fig. 4D). In addition, blue module and brown module were correlated with matrix score and immune score. Finally, we extracted 80 genes from the turquoise module for further analysis (Fig. 4E). We analyzed the protein interaction network (PPI) of these 80 genes and obtained that the hub1 gene was FN1. (Fig. 4 F)

#### The role of FN1 in thyroid cancer

**Fig. 5:**
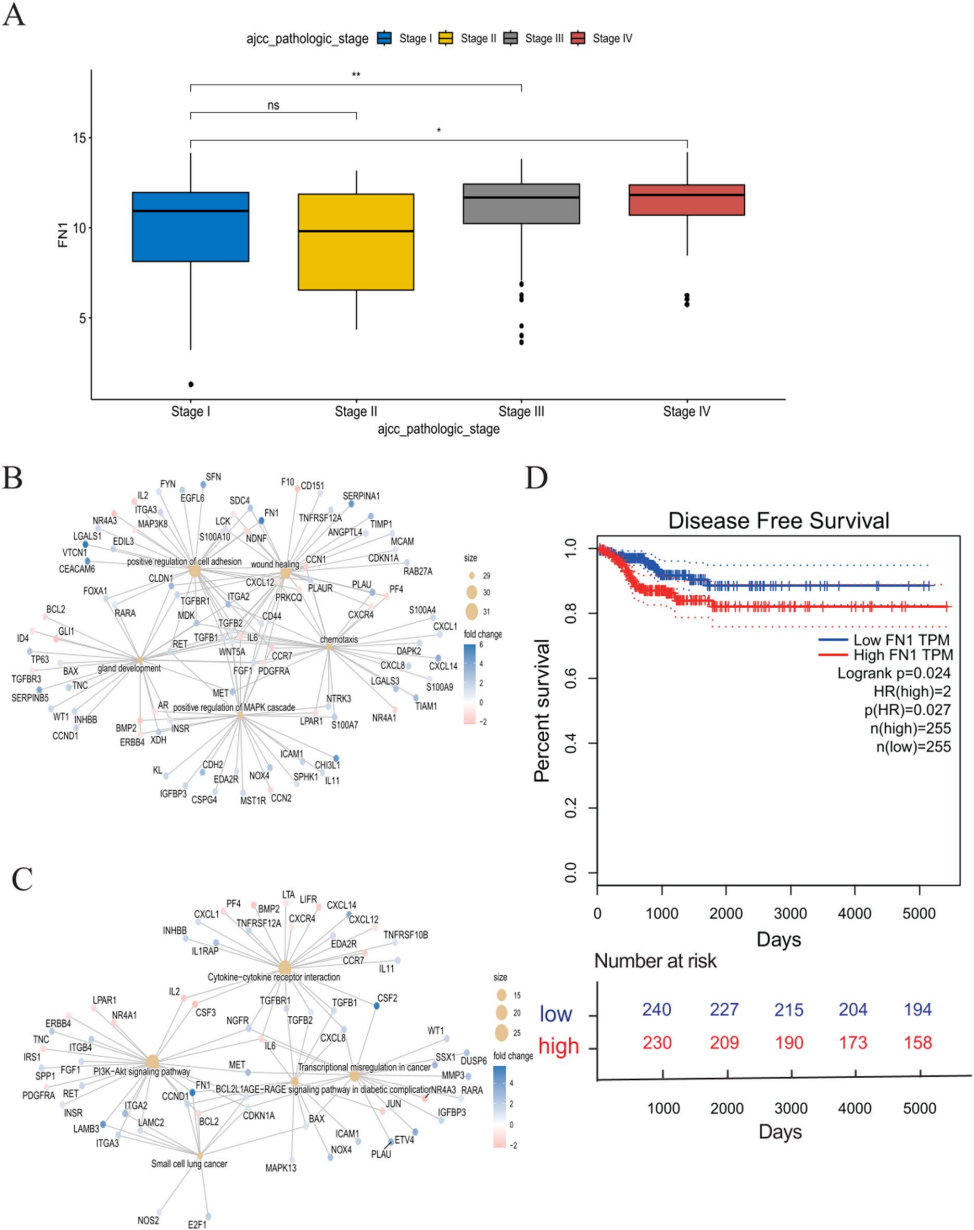
The role of FN1 in thyroid cancer. (**A**) Box plot of FN1 expression in different pathological stages (Stage I-IV), and the expression differences of each stage were compared (ns: no significant difference; *(*P* < 0.05), * *(*P* < 0.01): there are significant differences), stage I *n* = 120, stage II *n* = 80, stage III *n* = 45, stage IV *n* = 10. (**B**) The functional enrichment network of FN1-related genes. The node size is related to the number of genes involved. The color represents the trend of gene expression (blue is down-regulated, red is up-regulated). (**C**) The enrichment network of FN1-related pathways. The node size is related to the number of genes involved. The color represents the trend of gene expression. (**D**) Based on the Kaplan-Meier curve of disease-free survival based on the high and low groups of FN1 transcripts per million readings (TPM), the survival difference between the high and low FN1 TPM groups was significant (log-rank test *P* = 0.0024, hazard ratio HR = 1.32, high expression group n (high) = 255, low expression group n (low) = 255).

In order to explore the correlation between FN1 expression and tumor progression, we evaluated the expression level of FN1 in different AJCC pathological stages (I-IV) by box plot. Statistical analysis showed that there was no significant difference in FN1 expression between stage I and stage II (log2FC = -0.4, *P* = 0.522). In contrast, FN1 expression was significantly increased in stage III (log2FC = 1, *P* = 0.003) and stage IV (log2FC = 1.3, *P* = 0.017) compared with stage I. As shown in the figure, with the progress of tumor staging, FN1 expression showed a gradual upward trend (Fig. 5A). In addition, in thyroid cancer, Patients with high FN1 expression had significantly shorter disease-free survival than those with low expression, and FN1 was associated with the recurrence, progression and time span of death of thyroid cancer (Fig. 5D). Through GO and KEGG enrichment analysis, FN1 was mainly related to the biological process (BP) of positive regulation of cell adhesion in GO enrichment (Fig. 5B). In KEGG enrichment, FN1 was mainly related to cytokine − cytokine receptor interaction and PI3K / AKT pathway (Fig. 5C)^12,13^. FN1 As a core adhesion molecule in the extracellular matrix, FN1 is highly expressed in BRAF V600E-mutant papillary thyroid carcinoma and forms a key signaling axis with αvβ3 integrin to regulate tumor progression^14^. The present study shows that upregulation of FN1 enhances cell adhesion, activates anti-apoptotic pathways by binding to αvβ3 integrin, and modulates related apoptotic molecules. This inhibits apoptosis of cancer cells induced by matrix detachment and confers anoikis resistance. This mechanism is consistent with the results of GO analysis, as well as the known functions of fibronectin and established findings in thyroid cancer research.

#### Expression of FN1 in tumor microenvironment in TCGA-THCA

**Fig. 6:**
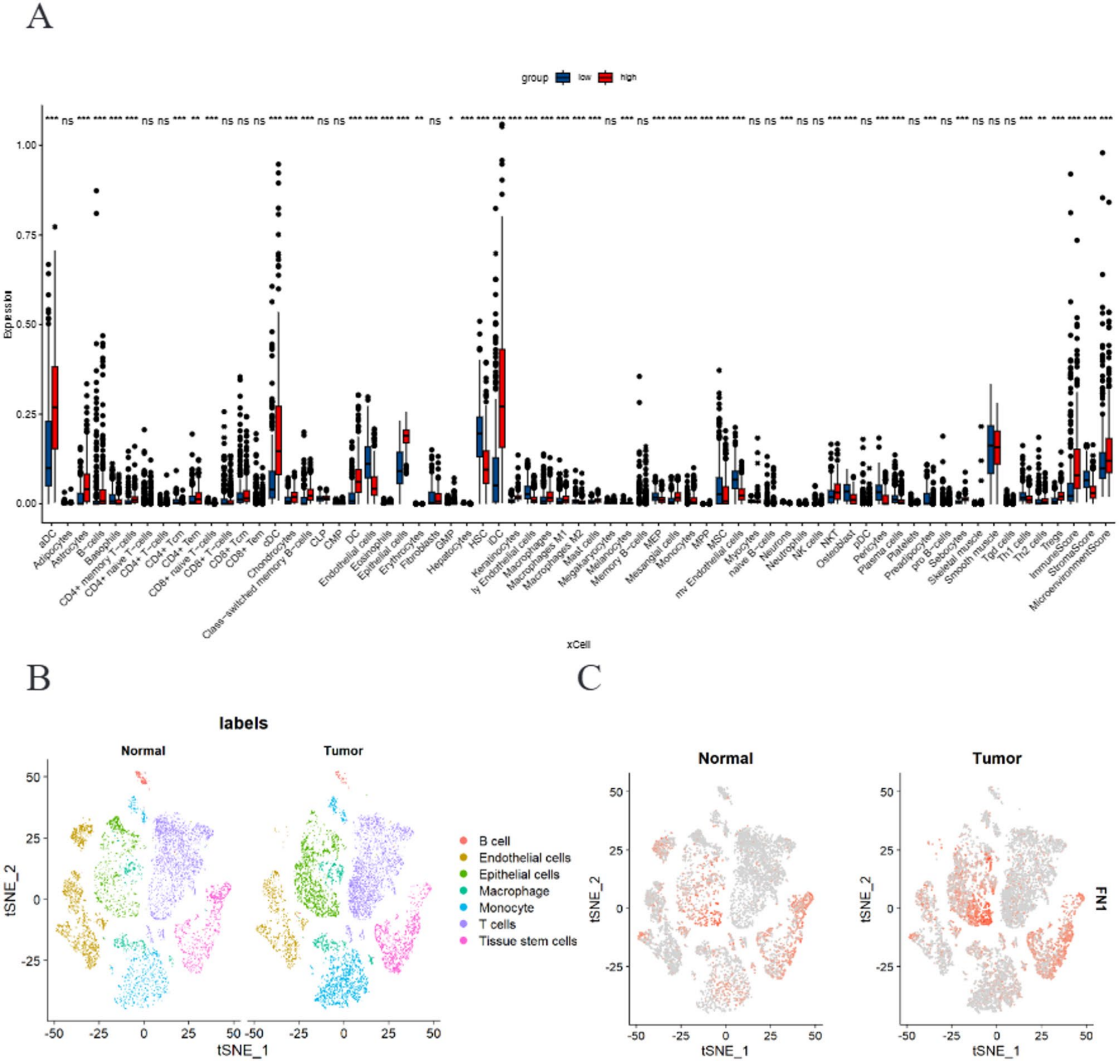
Expression of FN1 in tumor microenvironment in TCGA-THCA. (**A**) The proportion distribution of different cell types in FN1 low expression (blue) and FN1 high expression (red) samples in papillary thyroid cancer. (**B**) t-SNE dimensionality reduction maps of normal and tumor samples, stained by cell type (B cells, epithelial cells, macrophages, monocytes, tissue stem cells). (**C**) t-SNE dimensionality reduction maps of normal and tumor samples, showing the distribution of FN1 positive cells (red).

To further dissect the characteristics of immune cell infiltration in the tumor microenvironment (TME), we performed cell-type enrichment analysis on sample gene expression profiles using the xCell 2.0 algorithm (version 1.0.2). As shown in Fig. 6A, distinct immune infiltration-based clustering was observed among samples. Notably, the FN1 high-expression group exhibits unique immune microenvironment characteristics and shows hallmarks of T-cell response dysfunction. This feature clearly indicates a state of T-cell exhaustion, and this immune dysfunction impairs cytokine secretion and cytolytic activity, leading to the failure of immunosurveillance and the formation of an immune-suppressive TME that weakens effective anti-tumor immunity. These alterations collectively form an immune-excluded, immune-suppressive, and tumor-permissive microenvironment, which not only reduces the level of endogenous anti-tumor immunity but also may impair the body’s responsiveness to immune checkpoint blockade therapy. These results are highly consistent with the oncogenic role of FN1: FN1 overexpression induces T-cell dysfunction and exhaustion, actively shapes an immune-suppressive microenvironment, and ultimately promotes tumor immune evasion and malignant progression. To further dissect the cellular composition and gene expression dynamics between normal and tumor tissues, we performed t-distributed stochastic neighbor embedding (tSNE) analysis on single-cell RNA sequencing (scRNA-seq) data. As illustrated in Fig. 6B, major cell lineages (including B cells, endothelial cells, epithelial cells, macrophages, monocytes, T cells, and tissue stem cells, etc.) were clearly annotated. To clarify the expression pattern of FN1 (a key regulator of tumor progression), we visualized its transcript distribution in the tSNE dimensionality reduction plot (Fig. 6C), which allows direct assessment of the expression characteristics of FN1 across different cell populations in the two types of tissues.

### Cell experiments

#### Overexpression of FN1 in papillary thyroid cancer can promote migration, proliferation and invasion in vitro

**Fig. 7:**
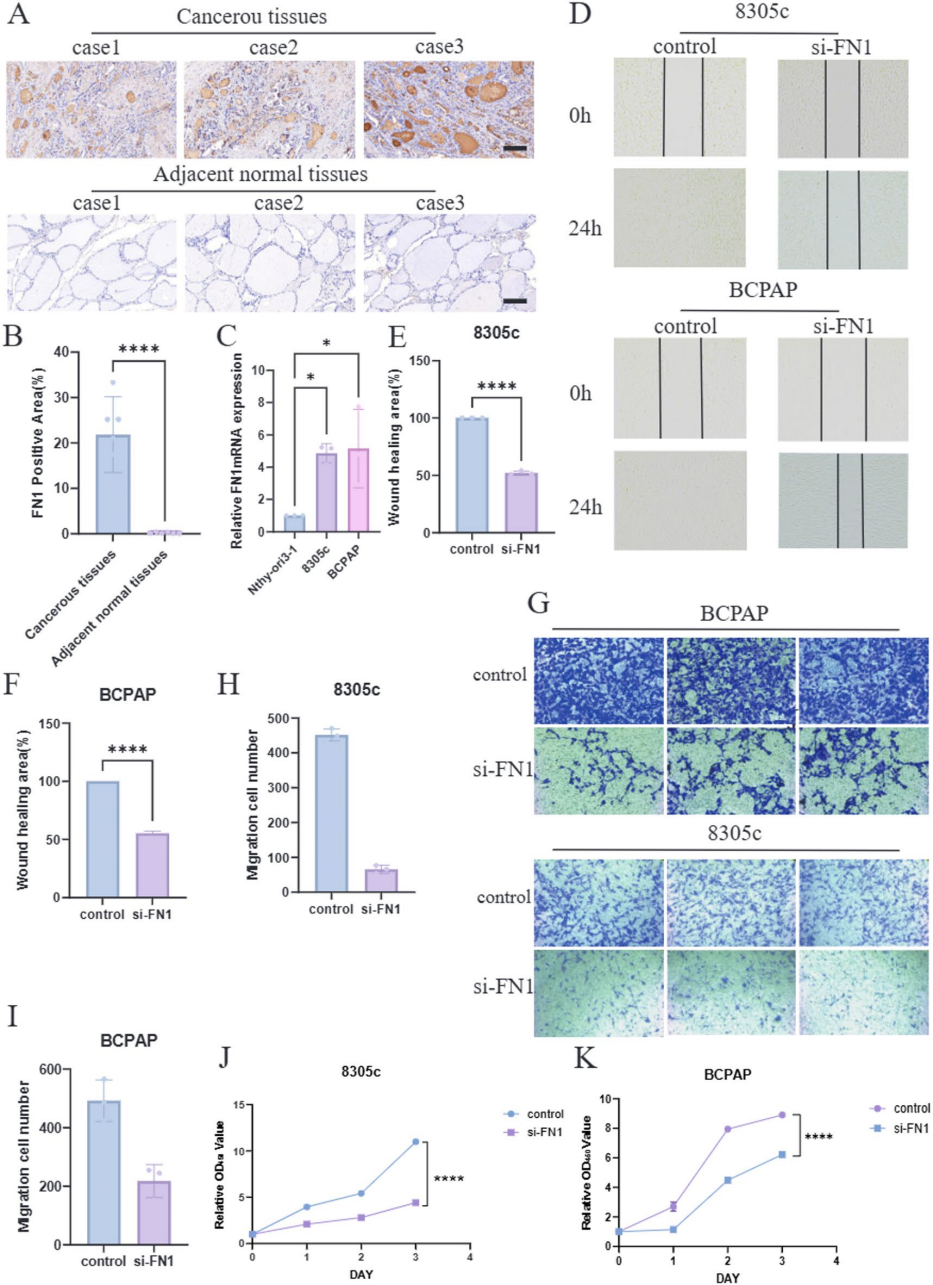
Overexpression of FN1 in papillary thyroid cancer can promote migration, proliferation and in*vasion in vitro* (**A**) Immunohistochemical staining results of fibronectin 1 (FN1) in cancer tissues and adjacent normal tissues. (**B**) Statistical analysis of the proportion of FN1 positive areas in cancer tissues and adjacent normal tissues (*P* < 0.0001). (**C**) Comparison of the relative expression of FN1 mRNA in Nthy-ori-3-1,8305 c and BCPAP cells (*P* < 0.05, *P* < 0.01). (**D**) Representative images of scratch test of 8305c and BCPAP cells (control group, FN1 interference group) at 0 h and 24 h. (E, F) Statistical analysis of 8305 c and BCPAP cell scratch healing area (*P* < 0.0001). (**G**) representative staining images of Transwell migration experiments of BCPAP and 8305c cells. (H-I) Statistical analysis of the number of migrated cells in 8305 c and BCPAP cells, respectively (J, K) CCK-8 test results of 8305 c and BCPAP cell proliferation (*P* < 0.0001).

We collected 33 cases of papillary thyroid carcinoma tissues for FN1 immunohistochemical staining, and found that the expression level of FN1 in papillary thyroid carcinoma was significantly higher than that in adjacent tissues (Fig. 7A, B). In addition, qRT-PCR suggested that FN1 was generally highly expressed in BCPAP and 8305c (Fig. 7C). In order to increase the control model of different differentiated subtypes of papillary thyroid cancer, we used 8305c and BCPAP cell lines for experiments. The expression of FN1 in thyroid cancer cell lines was knocked down by small interfering RNA, and the role of FN1 in the invasion and migration of thyroid cancer cells was evaluated by transwell and wound healing assays, indicating that the migration ability of thyroid cancer cells with FN1 knockdown was weakened, and the invasion activity was weakened (Fig. 7D–G). The expression of FN1 in thyroid cancer cell lines was knocked down by small interfering RNA, and the change of cell proliferation ability was detected by CCK-8 (Fig. 7J-K).

#### The anti-apoptotic ability and FN1 expression of thyroid carcinoma

**Fig. 8:**
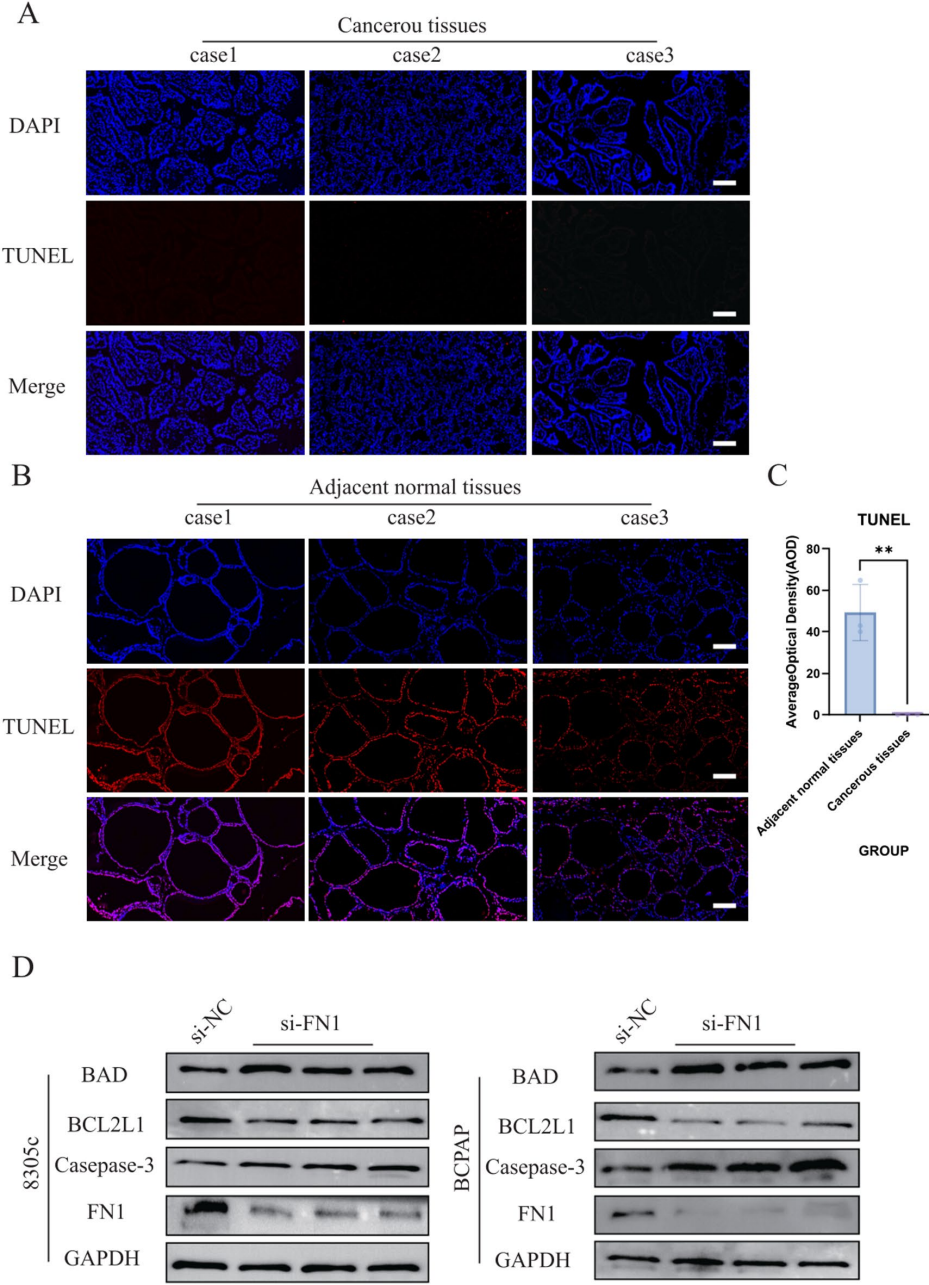
The anti-apoptotic ability and FN1 expression of thyroid carcinoma. (**A**) The results of immunofluorescence staining of papillary thyroid carcinoma (DAPI staining was blue, TUNEL labeled apoptotic cells were red, Merge was superimposed). (**B**) Immunofluorescence staining results in adjacent tissues of papillary thyroid carcinoma (the staining was the same as Fig. 7.A). (**C**) Statistical analysis of the average optical density (AOD) of TUNEL positive signals in normal tissues and cancer tissues (*P* < 0.001). (**D**) Western blot was used to detect the expression of FN1, Caspase-3, BAD and BCL2L1 protein in 8305c and BCPAP cells after interference with FN1 (si-FN1, 3 targets) and control (si-NC), GAPDH was used as internal reference.

To investigate the apoptotic characteristics of papillary thyroid cancer cells, we collected 16 cases of papillary thyroid carcinoma tissues from 16 distinct patients who underwent surgical resection in the Department of Thyroid Surgery, Affiliated Hospital of Guizhou Medical University, following the acquisition of ethical approval and written informed consent from all participants. Terminal deoxynucleotidyl transferase-mediated dUTP nick-end labeling (TUNEL) staining was performed to assess the apoptotic status of the tissues, and the results demonstrated that the apoptotic index of papillary thyroid carcinoma tissues was significantly lower than that of adjacent normal thyroid tissues (Fig. 8A–C). This finding indicates a reduced apoptotic potential in PTC tissues compared to their normal counterparts. In addition, when FN1 as knocked down, the expression of anti-apoptotic protein BCL2L1 in thyroid cancer cells decreased, while the expression of apoptosis-related proteins Caspase-3 and BAD increased, indicating that FN1 was closely related to the apoptosis ability of papillary thyroid cancer cells (Fig. 8D). Collectively, these results underscore that FN1 is closely associated with the apoptotic capacity of papillary thyroid cancer cells, and its expression may serve as a key regulatory factor in suppressing apoptosis in PTC.

## Discussion

Thyroid cancer, particularly papillary thyroid carcinoma (PTC), has emerged as one of the most prevalent endocrine malignancies worldwide, with its incidence steadily increasing in recent years. This rise is attributed to advancements in diagnostic imaging techniques, which have led to the detection of more cases, including those that may not have clinical significance. Despite the generally favorable prognosis associated with early-stage papillary thyroid cancer, a subset of patients experiences recurrence and metastasis, underscoring the need for a deeper understanding of the underlying molecular mechanisms that drive tumor progression. This complexity necessitates the identification of reliable biomarkers and therapeutic targets to enhance patient outcomes and treatment strategies.

In this study, we investigated the role of Fibronectin 1 (FN1) in papillary thyroid cancer, focusing on its implications for tumor malignant potential. Our findings elucidate the potential of FN1 as a pivotal player in promoting tumor cell survival and proliferation in the context of thyroid malignancies. As we delve into the implications of FN1 expression on tumor behavior and its correlation with clinical outcomes, this discussion will explore the intricate relationship between FN1 and various signaling pathways, highlighting its promise as a therapeutic target in advanced papillary thyroid cancer management. Building upon our systematic investigation of FN1 expression in papillary thyroid cancer, our data demonstrate that FN1 is markedly upregulated in both tumor tissues and cell lines, with its expression closely correlated to adverse clinical outcomes. These findings are congruent with several recent studies that revealed FN1 as a reliable prognostic biomarker in papillary thyroid cancer, with higher levels associated with advanced tumor stage, lymph node metastasis, and diminished patient survival rates^14,15^. Notably, immunohistochemical analyses have consistently identified FN1 overexpression across papillary, follicular, medullary, and undifferentiated subtypes, and such upregulation has been shown to associate with more aggressive pathological features^15,16^. However, our results further delineate that FN1 expression stratifies risk even among advanced cases, supporting its utility as a predictive marker for recurrence and progression. Of particular interest, while FN1 has been implicated in other epithelial malignancies as a marker of invasiveness and poor prognosis, our integrated approach underscores its specificity in papillary thyroid cancer, especially in relation to lymph node involvement and tumor stage, thereby adding a nuanced layer to its clinical interpretability^15^. In addition, the validity of the FN1-mediated anoikis resistance pathway is supported by two lines of evidence. First, this mechanism has been confirmed in multiple epithelial malignancies in gastric cancer, FN1 mediates anoikis resistance via activating the FAK/PI3K/Akt pathway and regulating the BCL2/Bax ratio, and FN1 knockdown enhances chemosensitivity, consistent with our finding that FN1 promotes PTC progression through the BCL2L1/BAD axis, revealing tumor subtype specificity in FN1-regulated apoptosis and providing insights for subtype-specific and combined therapies in PTC^17^. Another study focused on FN1’s splicing isoform, confirming that FN1-EDB promotes breast cancer lymphatic metastasis by activating the VEGF/VEGFR2 pathway to enhance anoikis resistance and lymphangiogenesis, with its targeted monoclonal antibody showing significant anti-tumor/anti-metastatic effects in mouse models^18^. These cross-cancer studies verify our findings, broaden understanding of FN1’s oncogenic function, and offer actionable strategies for clinical translation of FN1-related PTC research^19^. Second, this mechanism holds in thyroid cancer showed miR-142-3p regulates FN1 to activate the FAK/ERK/PI3K pathway, modulating PTC cell proliferation, invasion, apoptosis, and EMT^19^; demonstrated PLA2R1 competes with FN1 for ITGB1 binding to inhibit the FAK axis and EMT, with PLA2R1 knockout alleviating DTC in vivo growth^20^. In papillary thyroid cancer specifically, evidence suggests that FN1-driven focal adhesion signaling intersects with the regulation of apoptosis-related gene networks, conferring contributing to anti-apoptotic regulatory advantages and promoting metastatic competency^21^. These studies indicate FN1 is a core effector of PTC aggressiveness, mediating tumor progression via coordinating intracellular and microenvironmental signals. In summary, both cross-cancer and PTC-specific studies validate our findings, advance understanding of FN1’s oncogenic role, and support clinical translation of FN1-related PTC research.

The deployment of integrative bioinformatics analyses, including consensus clustering, WGCNA, and protein-protein interaction network construction, allowed us to robustly identify FN1 as a central anoikis-associated hub gene in papillary thyroid cancer. Previous studies utilizing machine learning and large-scale transcriptomic datasets have corroborated the centrality of FN1 among prognostic gene signatures, emphasizing its predictive power for disease progression and recurrence^22^. Our approach advances these observations by connecting FN1’s bioinformatic prominence with direct functional validation in cellular models. Comparative analyses of multiple datasets further demonstrate that FN1 consistently emerges as a top-ranked gene, irrespective of cohort composition or analytic platform^15,16^. This cross-validation enhances confidence in FN1’s universality as a molecular determinant of papillary thyroid cancer pathobiology, while also highlighting the utility of integrative data mining for prioritizing mechanistically relevant targets. Thus, the convergence of bioinformatics and experimental data substantiates FN1 as a bona fide central regulator within the tumor progression network.

Finally, regarding the translational implications of targeting FN1, our mechanistic findings position it as a promising therapeutic candidate for advanced or refractory papillary thyroid cancer. Preclinical work in papillary thyroid carcinoma has demonstrated that pharmacological disruption of the FN1/integrin axis—specifically using RGD-disintegrins to block FN1-αvβ3 interactions—can potently inhibit tumor cell proliferation and migration^15^. Additionally, studies targeting FN1 in combination with immune or kinase inhibitors have yielded synergistic anti-tumor effects, supporting a multifaceted therapeutic rationale^14^. Our study suggests that FN1 may have potential translational value in the clinical management of papillary thyroid carcinoma (PTC). As a stable and detectable extracellular matrix protein, FN1 could serve as a supplementary biomarker for diagnosis and prognosis using immunohistochemistry and liquid biopsy strategies. The combination of FN1 detection with artificial intelligence-assisted ultrasound may help improve the evaluation of indeterminate thyroid nodules. Meanwhile, integrating FN1 into multi-marker panels together with classic molecular, epigenetic, and immune markers may contribute to more accurate risk stratification and prognostic assessment. In terms of therapy, the FN1/Integrin/FAK/AKT signaling pathway might represent a potential target, especially for radioiodine-refractory PTC. Targeting FN1 could help enhance treatment sensitivity and may be combined with immune checkpoint inhibitors to modulate the tumor microenvironment. Patient-derived models may further support the future clinical translation of these approaches. Overall, FN1 shows potential as a complementary biomarker and candidate therapeutic target for PTC, which deserves further investigation in future clinical studies. While the development of FN1-targeted agents faces challenges related to specificity and off-target effects, our identification of its role in tumor cell migration, invasion and proliferation provides a strong mechanistic justification for continued drug development efforts. Importantly, the integration of FN1 as a biomarker for patient stratification could further enhance the efficacy and precision of future targeted therapies in papillary thyroid cancer.

This study has notable limitations that must be acknowledged. Firstly, the relatively small sample size used for experimental validation may limit the generalizability of the findings across diverse patient populations. Larger cohorts are essential to substantiate the role of FN1 in papillary thyroid cancer and to establish robust correlations with clinical outcomes. Secondly, due to the technical bottleneck in suspension culture of adherent-dependent solid tumor cells, this study did not establish a poly (2-hydroxyethyl methacrylate) (Poly-HEMA) - coated suspension culture model to directly verify FN1’s anoikis resistance function; future studies should therefore optimize the cell culture system and perform direct suspension experiments to more intuitively confirm FN1’s role in PTC anoikis resistance. Finally, while bioinformatics analyses provide insightful correlations, the inherent variability in the datasets, such as batch effects and heterogeneity among samples, could introduce biases that affect the reliability of FN1 as a therapeutic target. Future research should aim to include larger, more diverse clinical samples and implement rigorous validation protocols to strengthen the conclusions drawn.

In summary, this research elucidates the critical role of FN1 in papillary thyroid cancer, particularly its function in regulating cell proliferation, migration, invasion and apoptosis, thereby highlighting its potential as a therapeutic target. The innovative integration of bioinformatics analysis with experimental validation represents a significant advancement in understanding the molecular mechanisms underpinning papillary thyroid cancer progression. Future studies should focus on developing targeted therapies against FN1 and explore its utility in combination treatment strategies, paving the way for improved clinical interventions in advanced or metastatic papillary thyroid cancer cases.

## Supplementary Information

Supplementary Material 1.

Supplementary Material 2.

Supplementary Material 3.

## Data Availability

All data and materials supporting the conclusions of this article are publicly available or can be obtained through reasonable requests, with detailed access information and availability specifications provided below. Public Database Data TCGA-THCA Dataset: RNA-sequencing data (FPKM format, normalized to TPM) and matched clinical metadata (pathological stage, TNM classification, survival outcomes) of thyroid carcinoma (THCA) were retrieved from The Cancer Genome Atlas (TCGA) database ( [https://portal.gdc.cancer.gov/](https:/portal.gdc.cancer.gov) ). The dataset initially included 510 primary papillary thyroid cancer samples and 58 adjacent non-tumor thyroid samples; after excluding samples with incomplete clinical information, 498 qualified cancer samples were used for subsequent analyses. No additional access restrictions apply beyond TCGA’s public access policies.Single-Cell RNA-Seq (scRNA-seq) Dataset: The public scRNA-seq dataset (GSE241184) was downloaded from the Gene Expression Omnibus (GEO) database ( [https://www.ncbi.nlm.nih.gov/geo/](https:/www.ncbi.nlm.nih.gov/geo) ), containing 16,897 cells derived from 1 patient with papillary thyroid carcinoma (PTC) and lymph node metastasis. Raw data were processed with quality control (mitochondrial gene content < 5%, unique gene counts > 200) and normalized using the SCTransform function in Seurat. The dataset can be accessed via GEO with the accession number GSE241184.GEPIA Database Validation Data: Differential expression of FN1 between papillary thyroid cancer and normal tissues, and its association with disease-free survival (DFS), were validated using the GEPIA database ( [http://gepia.cancer-pku.cn/](http:/gepia.cancer-pku.cn) ). Analyses relied on GEPIA’s built-in TCGA and GTEx datasets, and results can be reproduced via the database’s “Expression DIY” and “Survival Analysis” modules with default parameters (log-rank test, *P* < 0.05).Anoikis-Related Genes (ARGs): A total of 908 ARGs were obtained from the GeneCards database ( [https://www.genecards.org/](https:/www.genecards.org) ) using “anoikis” as the search term; genes with a “Relevance Score” ≥ 50 were retained for analysis. These genes are publicly accessible through GeneCards without restrictions.Clinical Sample DataClinical tissue specimens (32 papillary papillary thyroid cancer tissues, 32 peritumoral tissues, and 1 lymph node tissue) were collected from 33 PTC patients at the Affiliated Hospital of Guizhou Medical University (November 2024–August 2025), with ethical approval from the hospital’s Ethics Review Committee (Approval No.: GMU2024-452) and written informed consent from all participants. Due to privacy protection requirements for human subject data, raw clinical metadata (e.g., patient demographics, full survival records) are not publicly available. Requests for access to de-identified clinical data can be submitted to the corresponding author (Hui Ye, E-mail: 1442619877@qq.com) and will be granted upon review of the request against ethical and legal guidelines. Supplementary DataDue to the large memory size of all raw data, they cannot be uploaded to your journal’s system. All raw data have been uploaded to Zenodo ( [https://zenodo.org/](https:/zenodo.org) ). You may request access to all my raw data through this website.

## Abbreviations

AJCC: American joint committee on cancer
AKT: Protein kinase B
AOD: Average optical density
ARGs: Anoikis-related genes
BP: Biological processes
CC: Cellular components
CCK-8: Cell counting kit-8
DE-ARGs: Differentially expressed anoikis-related genes
DEGs: Differentially expressed genes
DFS: Disease-free survival
DMEM: Dulbecco’s modified eagle medium
ECM: Extracellular matrix
EDB: Extra domain B
ERK: Extracellular signal-regulated kinase
FAK: Focal adhesion kinase
FBS: Fetal bovine serum
FDR: False discovery rate
FN1: Fibronectin 1
FPKM: Fragments per kilobase of exon per million fragments mapped
FTC: Follicular thyroid carcinoma
GEO: Gene expression omnibus
GO: Gene ontology
HR: Hazard ratio
IHC: Immunohistochemistry
ITGB1: Integrin beta 1
KEGG: Kyoto encyclopedia of genes and genomes
MAPK: Mitogen-activated protein kinase
MF: Molecular functions
MTC: Medullary thyroid carcinoma
NC: Negative control
PI3K: Phosphatidylinositol 3-kinase
PLA2R1: Phospholipase A2 receptor 1
PPI: Protein-Protein interaction
PTC: Papillary thyroid carcinoma
qRT-PCR: Quantitative reverse transcription-polymerase chain reaction
scRNA-seq: Single-cell RNA sequencing
siRNA: Small interfering RNA
TCGA: The cancer genome atlas
TIMPs: Tissue inhibitors of metalloproteinases
TPM: Transcripts per million
tSNE: t-Distributed stochastic neighbor embedding
VEGF: Vascular endothelial growth factor
WGCNA: Weighted gene co-expression network analysis
