# Supplementary material for: The mechanism of fibronectin 1 promoting papillary thyroid cancer progression by regulating anoikis resistance
Source: Sci Rep. 2026 Apr 19;16:17857. doi: 10.1038/s41598-026-43495-8 (PMC13249844; doi:10.1038/s41598-026-43495-8)
Supplement: Supplementary file 1 — Supplementary Material 1. [file 41598_2026_43495_MOESM1_ESM.docx]

Due to the large memory size of all raw data, they cannot be uploaded to your journal's system. All raw data have been uploaded to Zenodo (<https://zenodo.org/>), and my data DOI is <https://doi.org/10.5281/zenodo.17499354>. You may request access to all my raw data through this website and the provided DOI.
