# Supplementary material for: The mechanism of fibronectin 1 promoting papillary thyroid cancer progression by regulating anoikis resistance
Source: Sci Rep. 2026 Apr 19;16:17857. doi: 10.1038/s41598-026-43495-8 (PMC13249844; doi:10.1038/s41598-026-43495-8)
Supplement: Supplementary file 2 — Supplementary Material 2. [file 41598_2026_43495_MOESM2_ESM.pdf]

1. Authorization for the use of the KEGG pathway database

In accordance with the requirements of your journal and KEGG's authorization guidelines, I have completed the application for KEGG usage permission strictly following the procedures outlined in your previous correspondence. Additionally, the relevant KEGG citations have been properly included in the manuscript as required.

Due to KEGG's internal processing protocols, the official authorization document is still pending and is expected to take a few working days to finalize. Rest assured that upon receiving the permission from KEGG, I will immediately upload it to your journal's submission system or forward it to your designated email address without delay.

I sincerely apologize for any inconvenience this may cause to your editorial workflow and kindly request your understanding.

2. Specifications and requirements for gels/blots

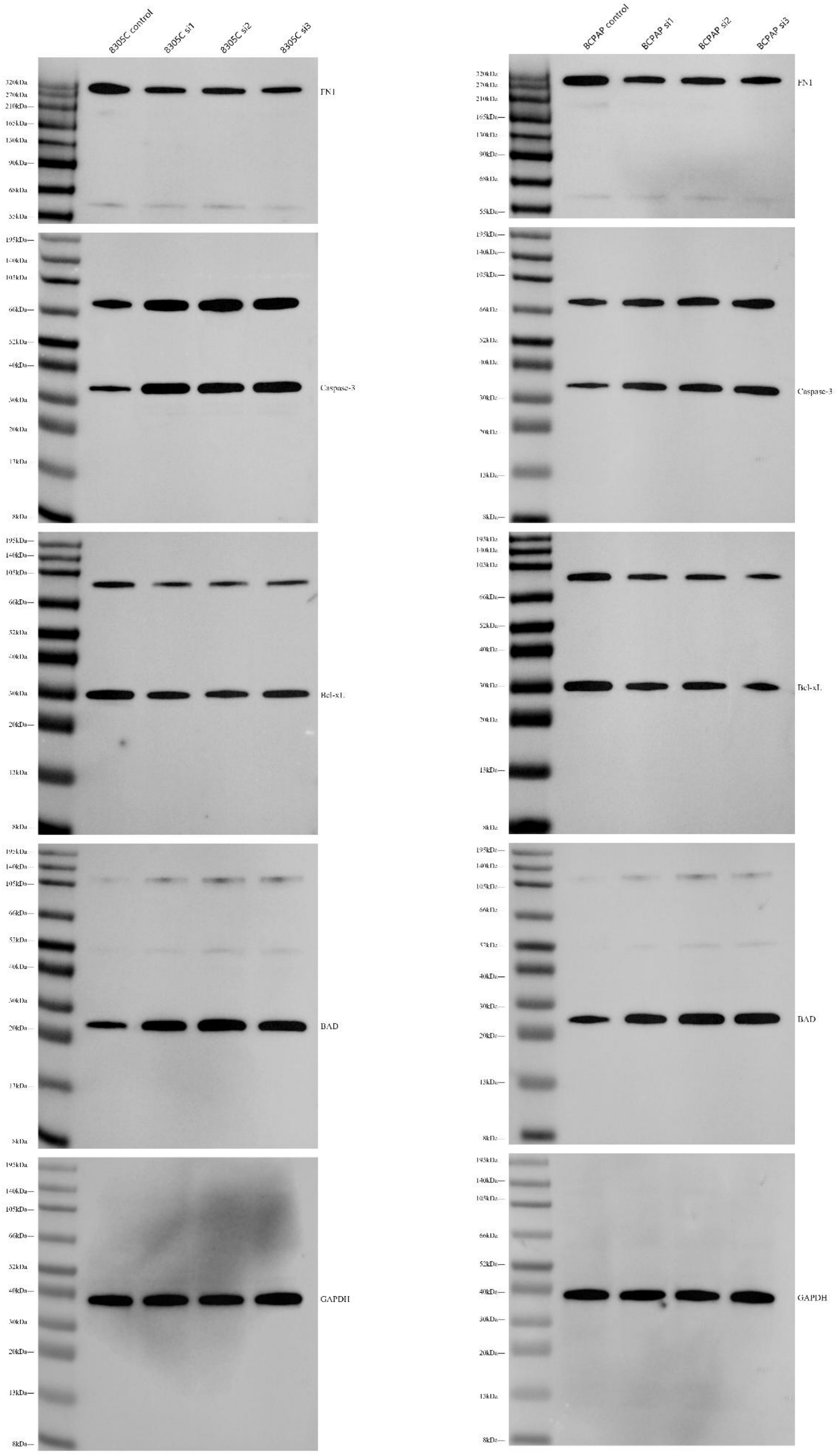
8305C control
8305C si1
8305C si2
8305C si3
BCPAP control
BCPAP si1
BCPAP si2
BCPAP si3
FN1
Caspase-3
Bcl-xL
BAD
GAPDH
