# Supplementary figures and images for: The mechanism of fibronectin 1 promoting papillary thyroid cancer progression by regulating anoikis resistance
Source: Sci Rep. 2026 Apr 19;16:17857. doi: 10.1038/s41598-026-43495-8 (PMC13249844; doi:10.1038/s41598-026-43495-8)

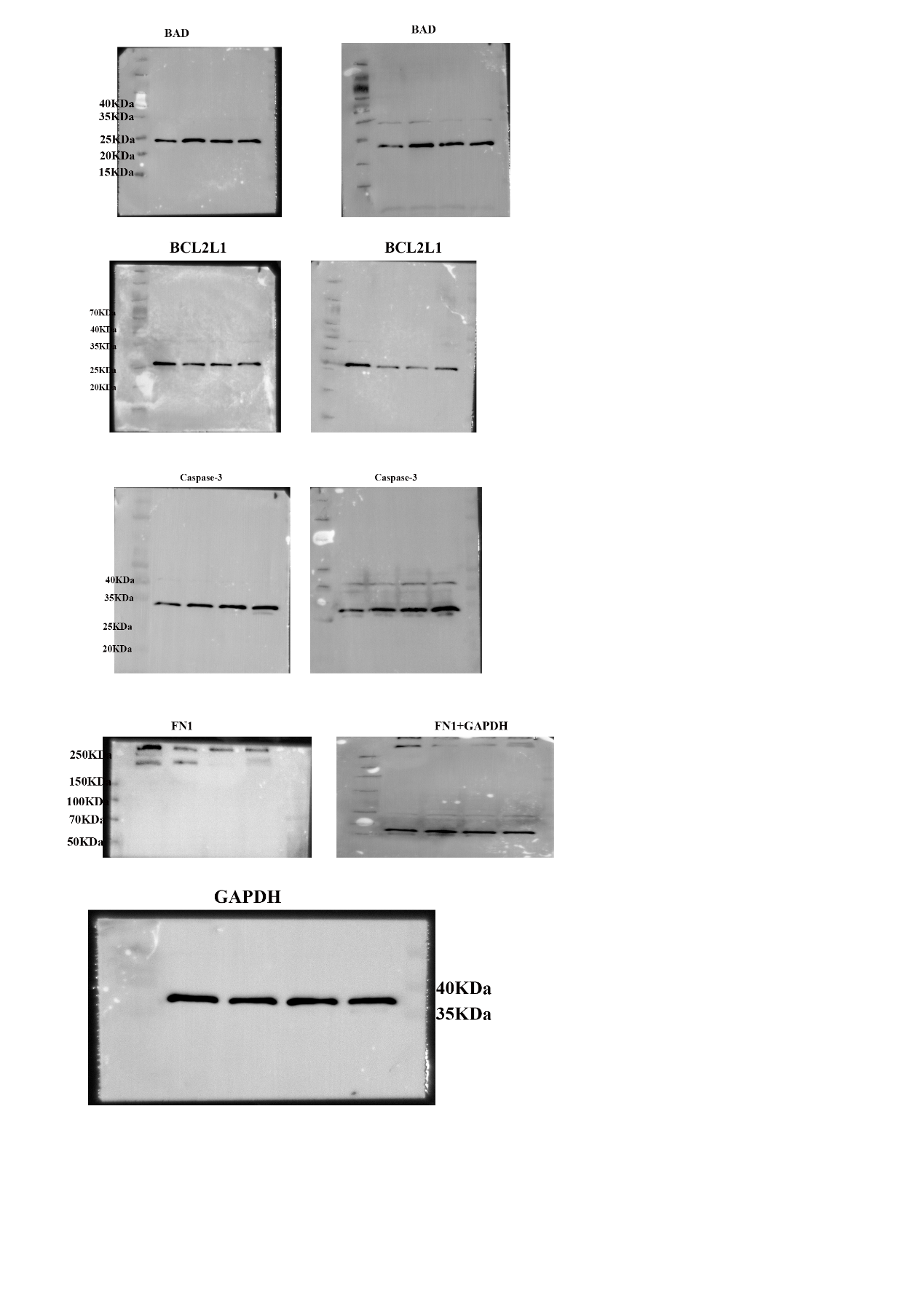

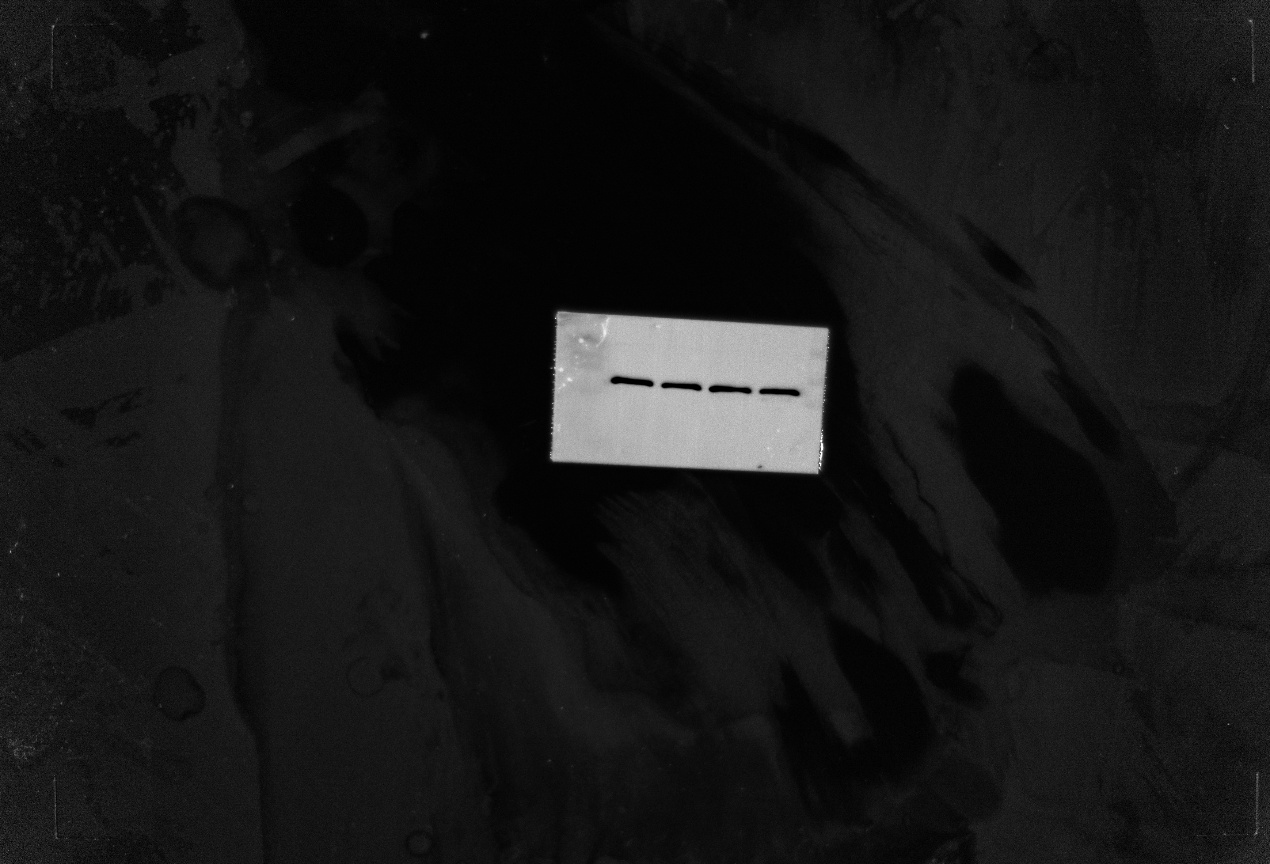


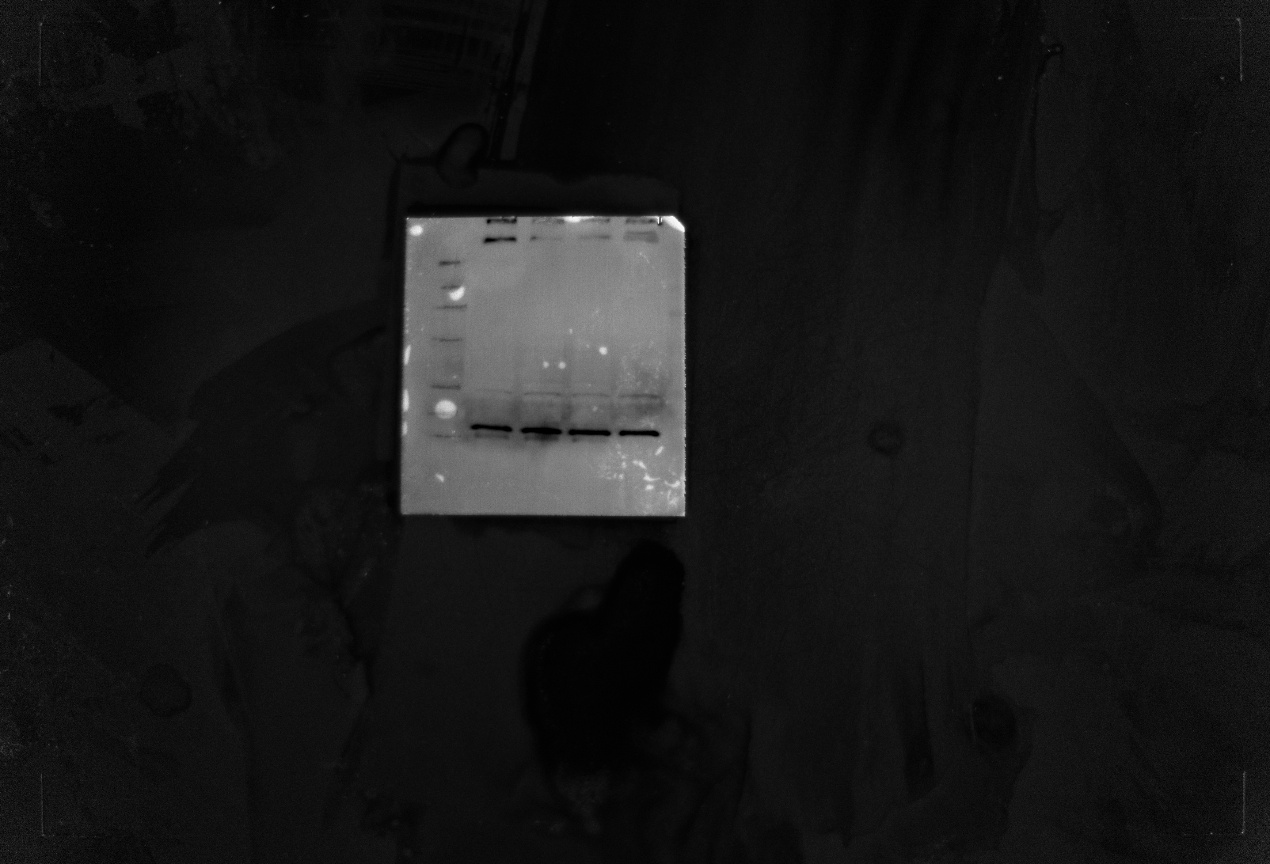

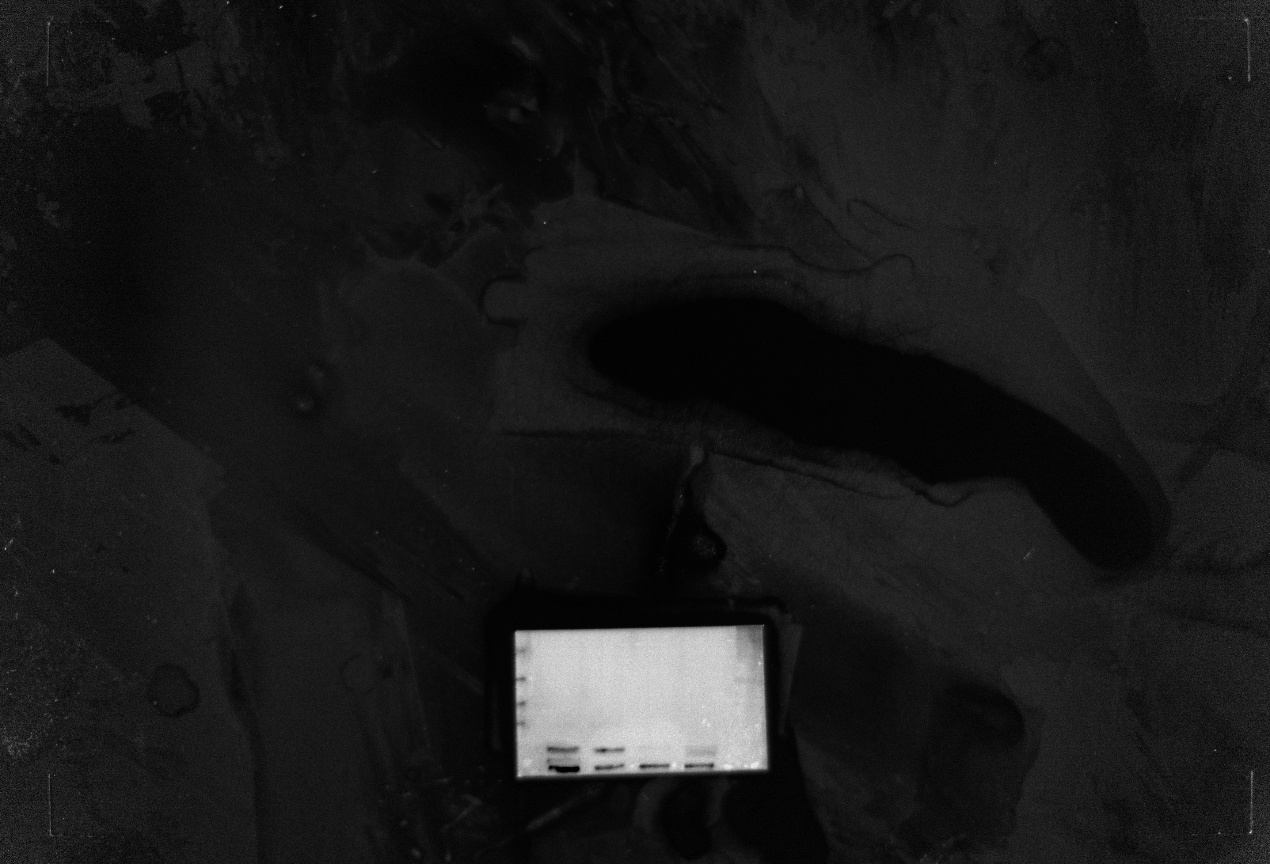

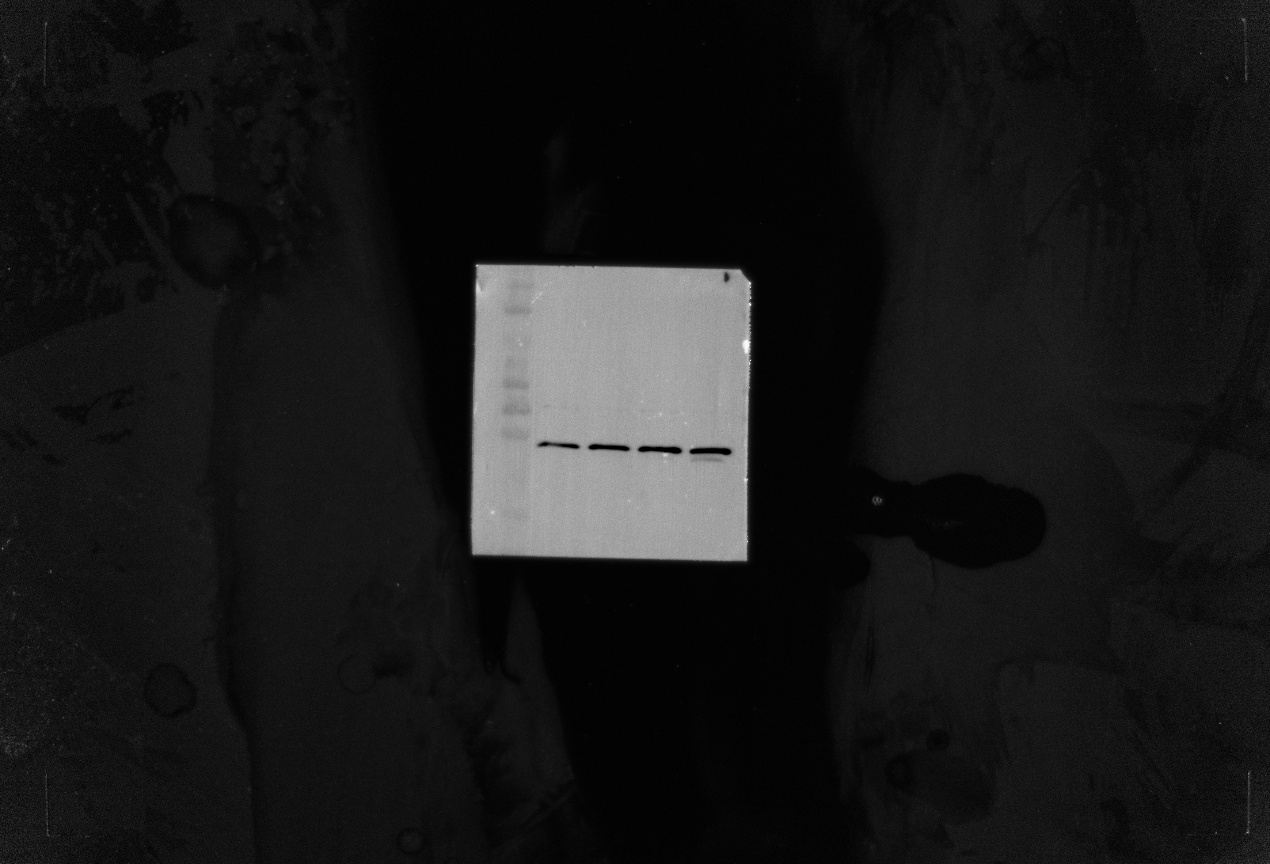

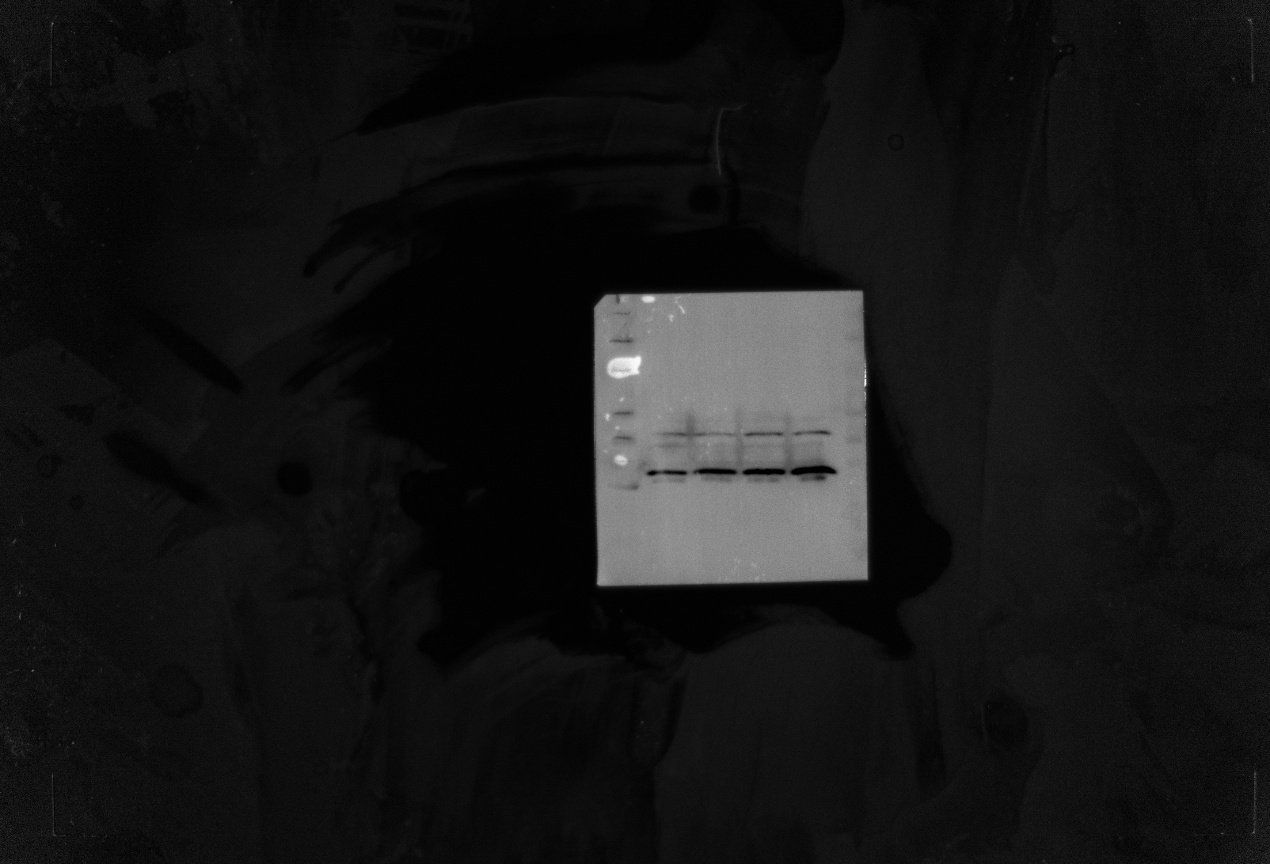

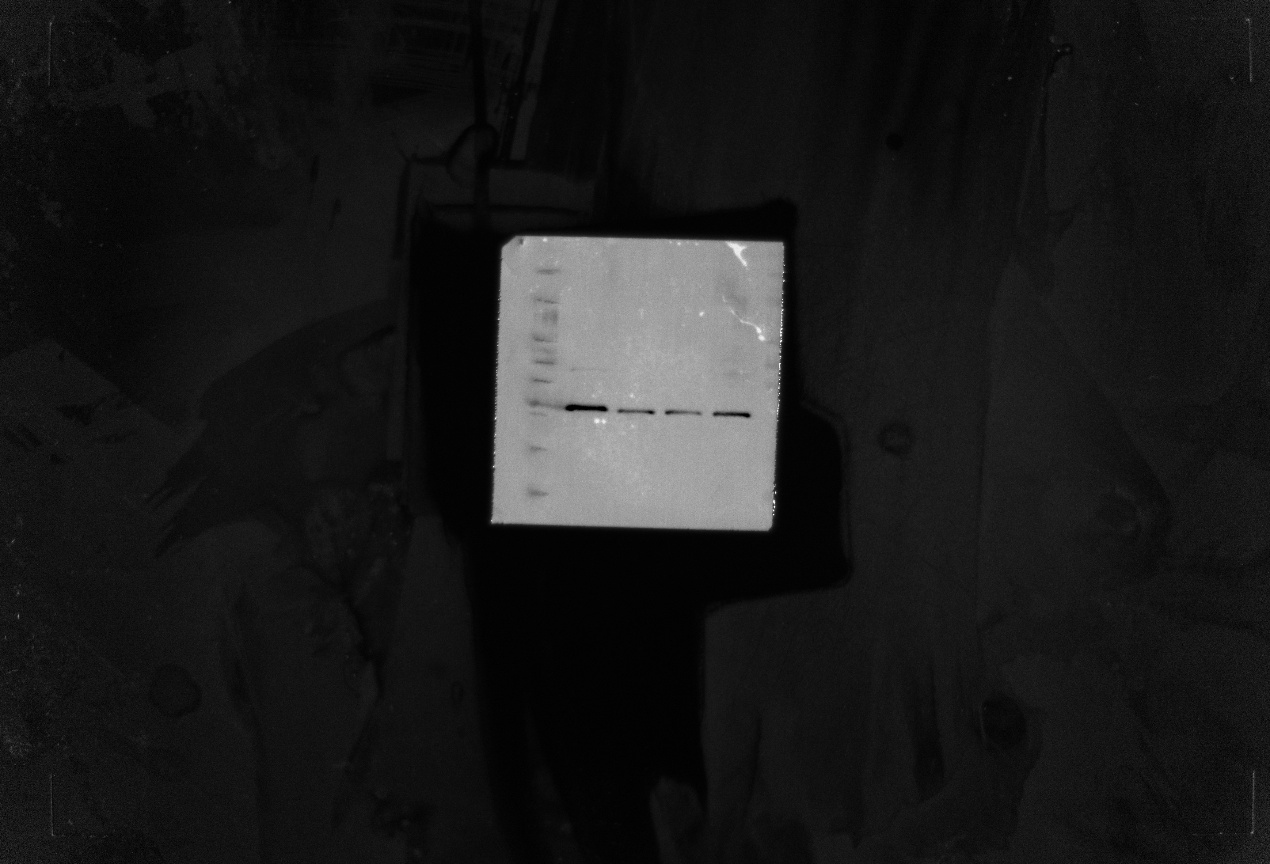

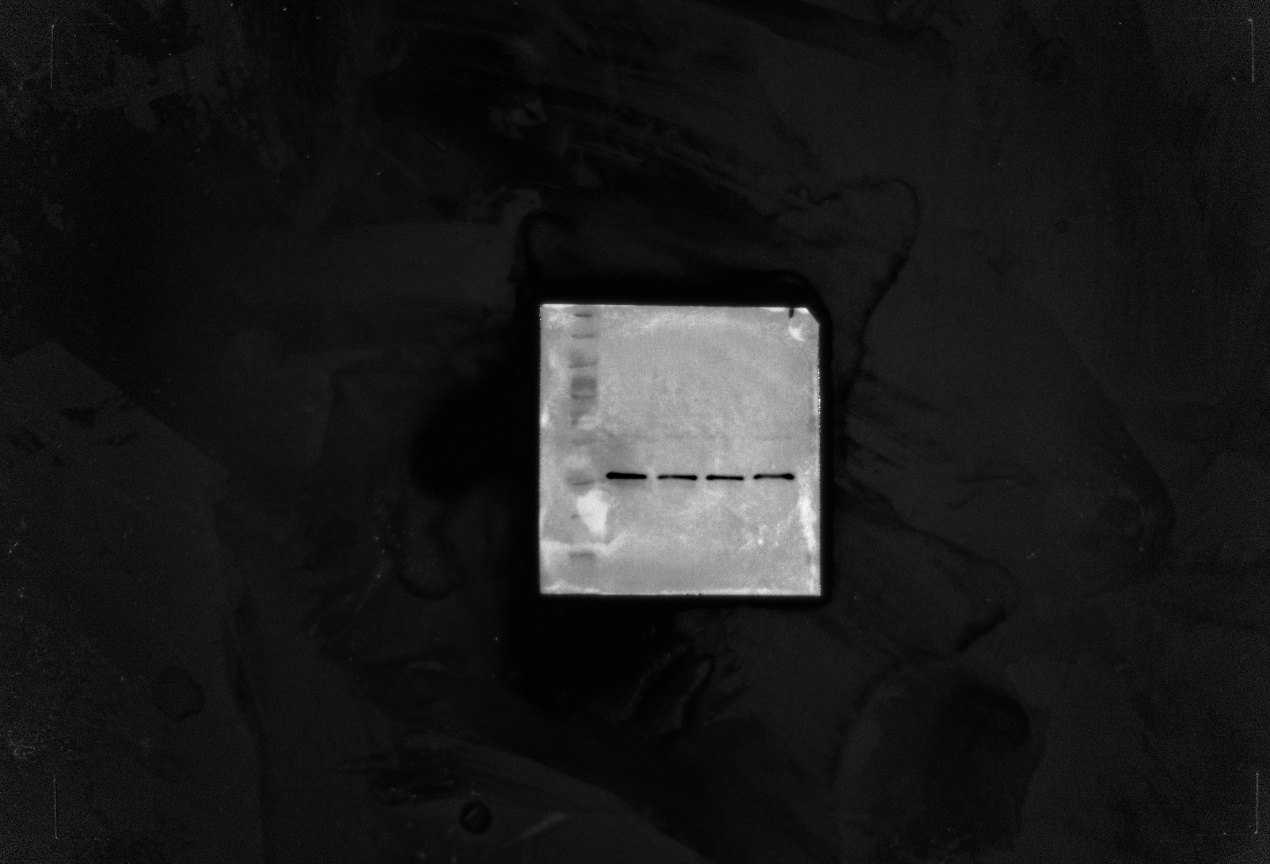

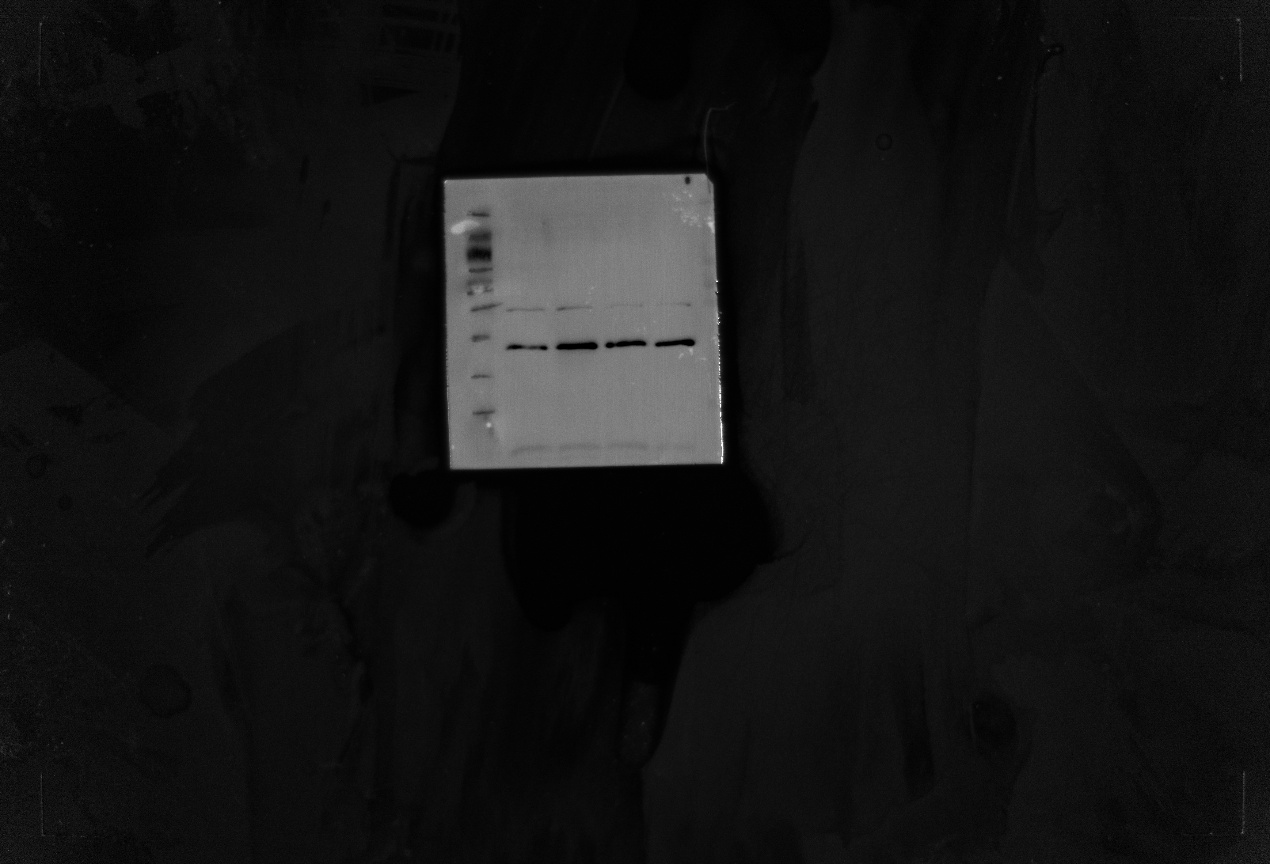

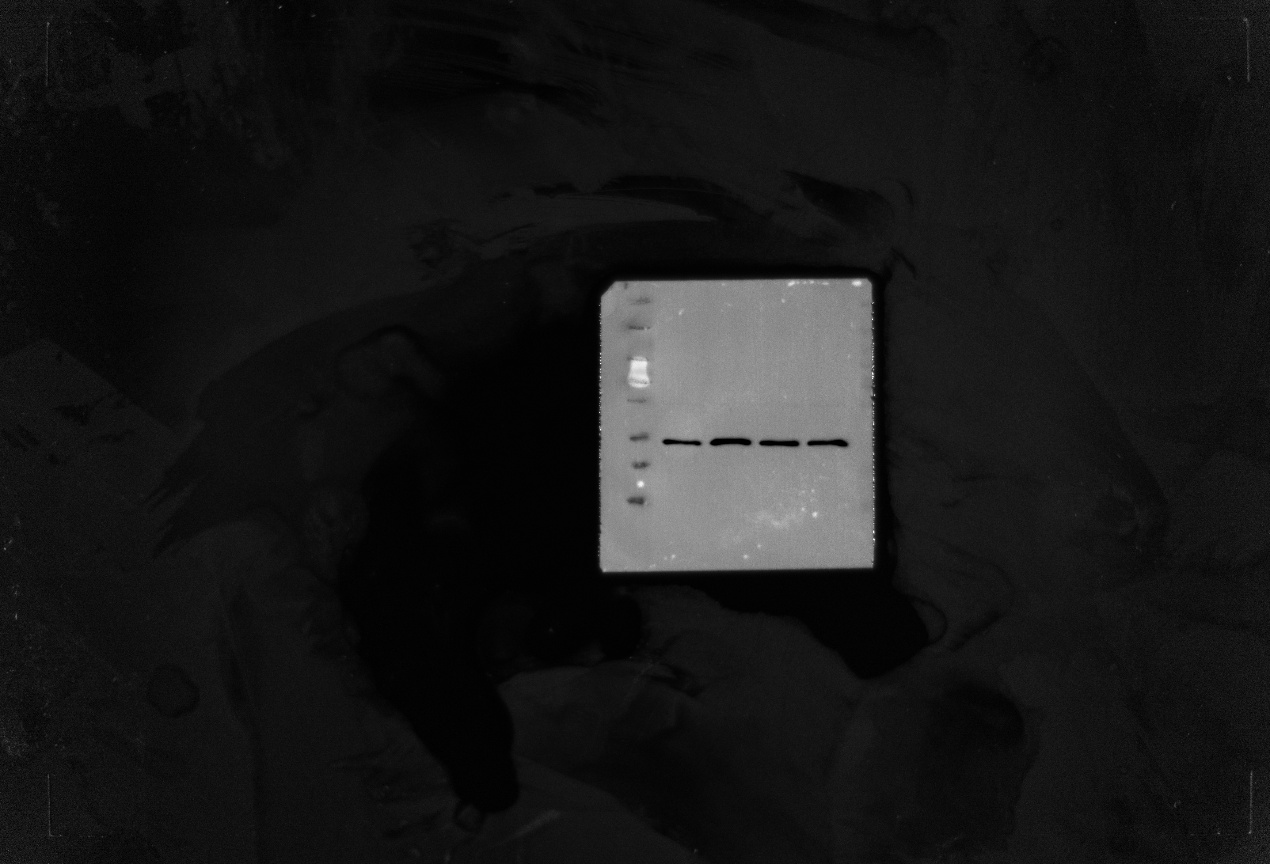

Supplement: Supplementary file 3 — Supplementary Material 3. [file 41598_2026_43495_MOESM3_ESM.docx]
